# Supplementary material for: Oligoprotein type I interferon signatures, but not TREX1 variants, increase risk of systemic lupus erythematosus in UK Biobank
Source: Nat Commun. 2026 Jan 27;17:1073. doi: 10.1038/s41467-025-67832-z (PMC12852752; doi:10.1038/s41467-025-67832-z)
Supplement: Supplementary file 1 — Supplementary Information [file 41467_2025_67832_MOESM1_ESM.pdf]

SUPPLEMENTAL MATERIAL

Oligoprotein type I interferon signatures, but not *TREX1* variants, increase risk of systemic lupus erythematosus in UK Biobank

Authors and affiliations:

Bastien Rioux[1,2], Sarah McGlasson[1,2], Deborah Forbes[1,2], Katy R. Reid[1,2], Anna Klingseisen[1,2], Joe Berry[3], Neeraj Dhaun[4], Wan Fai Ng[3,5,6], William Whiteley[1,7], David P. J. Hunt[1,2]

[1] Institute for Neuroscience and Cardiovascular Research, University of Edinburgh, Edinburgh, United Kingdom

[2] UK Dementia Research Institute at Edinburgh, Edinburgh, United Kingdom

[3] Newcastle University, Newcastle upon Tyne, United Kingdom

[4] Edinburgh Kidney Research Group, University/BHF Centre for Cardiovascular Science, The Queen's Medical Research Institute, University of Edinburgh, Edinburgh, United Kingdom

[5] University College Cork, Cork, Ireland

[6] Department of Rheumatology, Cambridge University Hospitals, Cambridge, United Kingdom

[7] British Heart Foundation Data Science Centre, Health Data Research UK, London, United Kingdom

Table of Contents

SUPPLEMENTARY FIGURES ... 3

Supplementary Figure 1. Microscopic analysis of the effect of *TREX1* variants on EGFP-TREX1 protein localisation. ... 3
Supplementary Figure 2. UpSet plot of sources of diagnosis for lupus and Sjogren disease in UK Biobank. ... 4
Supplementary Figure 3. Derivation and validation of the Markers of type I Interferon Response in Olink (MIRO) score. ... 5
Supplementary Figure 4. Clinical associations of reported disease-causing *TREX1* variants. ... 6
Supplementary Figure 5. Detection of oligoprotein interferon signatures before diagnosis of lupus in UK Biobank. ... 7
Supplementary Figure 6. Density plots of components of the MIRO score by (A) SLE/SjD status and (B) *TREX1* genotype in UK Biobank. ... 8
Supplementary Figure 7. Replication of analyses in genetically unrelated UK Biobank participants. ... 9
Supplementary Figure 8. Funnel plot for *TREX1*-lupus association studies included in the review. ... 10

SUPPLEMENTARY TABLES ... 11

Supplementary Table 1. Demographics of participants by *TREX1* genotype. ... 11
Supplementary Table 2. Sources of diagnosis for systemic lupus erythematosus. ... 12
Supplementary Table 3. Demographics of participants by systemic lupus erythematosus status and data availability. ... 13
Supplementary Table 4. Sources of diagnosis for systemic lupus erythematosus and Sjogren disease. ... 14
Supplementary Table 5. Association of reported *TREX1* risk variants with systemic lupus erythematosus and Sjogren disease in UK Biobank using SAIGE-GENE+. ... 15
Supplementary Table 6. Definition of expanded clinical and radiological phenotypes. ... 16
Supplementary Table 7. Mapping of phenotypes that were not defined from UK Biobank first occurrence fields. ... 20
Supplementary Table 8. Description of the 84 disease-causing *TREX1* variants included. ... 25
Supplementary Table 9. Association of MIRO scores at baseline with incident diagnoses in UK Biobank. ... 32
Supplementary Table 10. Description of the 35 reported disease-causing *TREX1* variants observed in UK Biobank. ... 33
Supplementary Table 11. Association of reported disease-causing *TREX1* variants with outcomes in UK Biobank using SAIGE-GENE+. ... 36
Supplementary Table 12. Descriptive analysis of systemic lupus erythematosus/Sjogren disease cases and type I interferon signature by *TREX1* genotype. ... 38
Supplementary Table 13. Search strategy in Ovid EMBASE and Ovid MEDLINE. ... 39
Supplementary Table 14. Description of studies included in the systematic review. ... 40
Supplementary Table 15. Risk of bias assessment (Q-Genie) for studies included in the systematic review. ... 42
Supplementary Table 16. Weights for each protein included in the MIRO score defined from their β coefficient in the penalized logistic regression. ... 43

SUPPLEMENTARY NOTES ... 44

Supplementary Note 1. Checklist for the STrengthening the REporting of Genetic Association studies (STREGA) reporting recommendations, extended from STROBE Statement. ... 44
Supplementary Note 2. Checklist for the Preferred Reporting Items for Systematic Reviews and Meta-analyses (PRISMA) statement. ... 51

SUPPLEMENTARY REFERENCES ... 54

SUPPLEMENTARY FIGURES

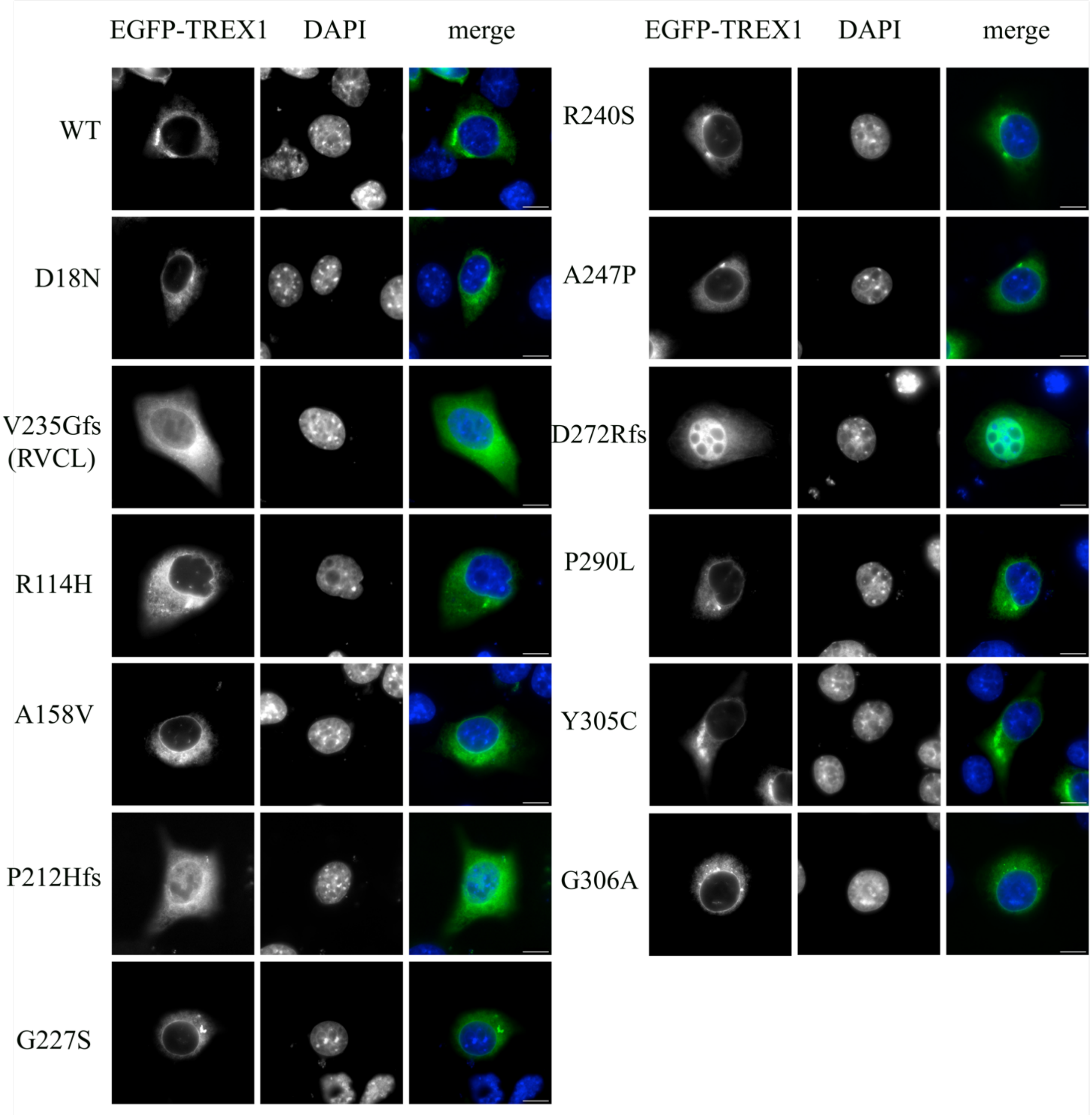

Supplementary Figure 1. Microscopic analysis of the effect of *TREX1* variants on EGFP-TREX1 protein localisation.

EGFP-TREX1 variants were transiently expressed in *Trex1*$^{-/-}$ MEFs. Cells were fixed and co-stained with DAPI (4′,6-diamidino-2-phenylindole nuclear DNA stain). Images were taken at 63X. Scale bar=10 microns.

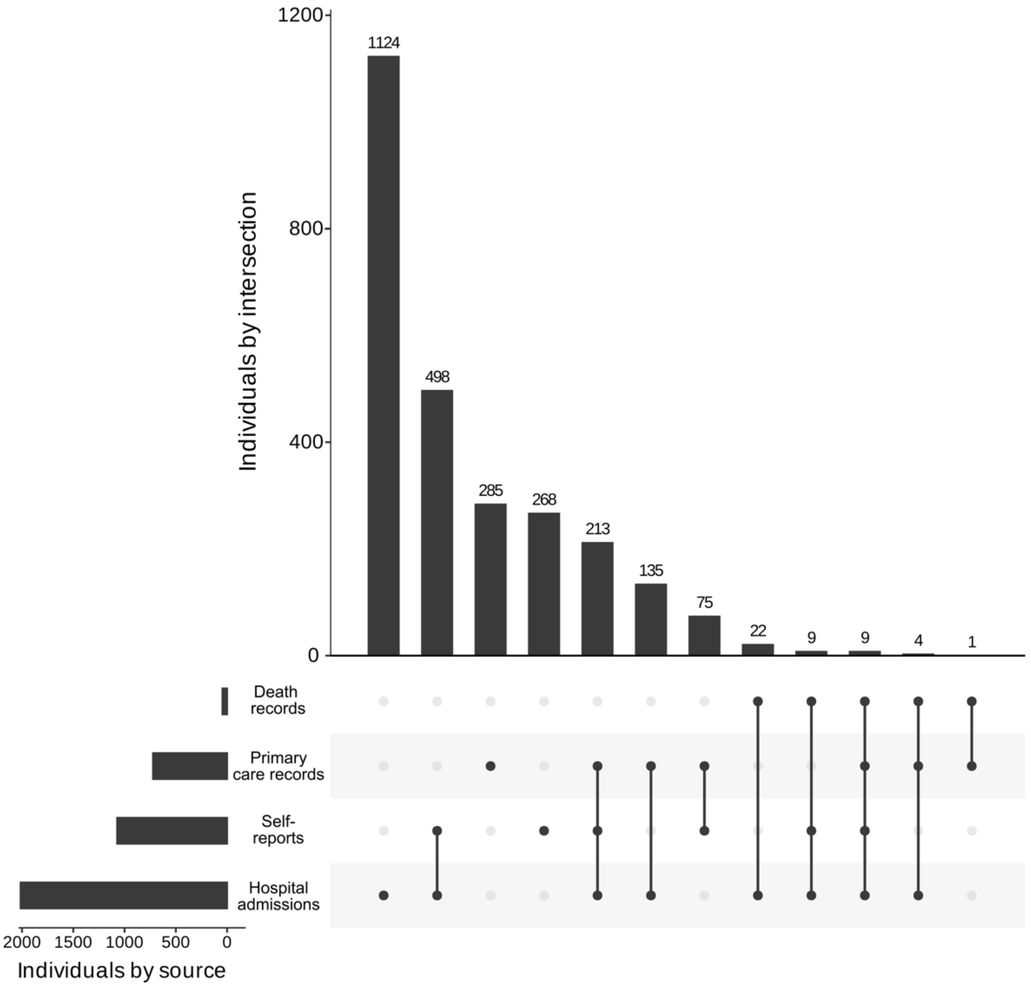

Supplementary Figure 2. UpSet plot of sources of diagnosis for lupus and Sjogren disease in UK Biobank.

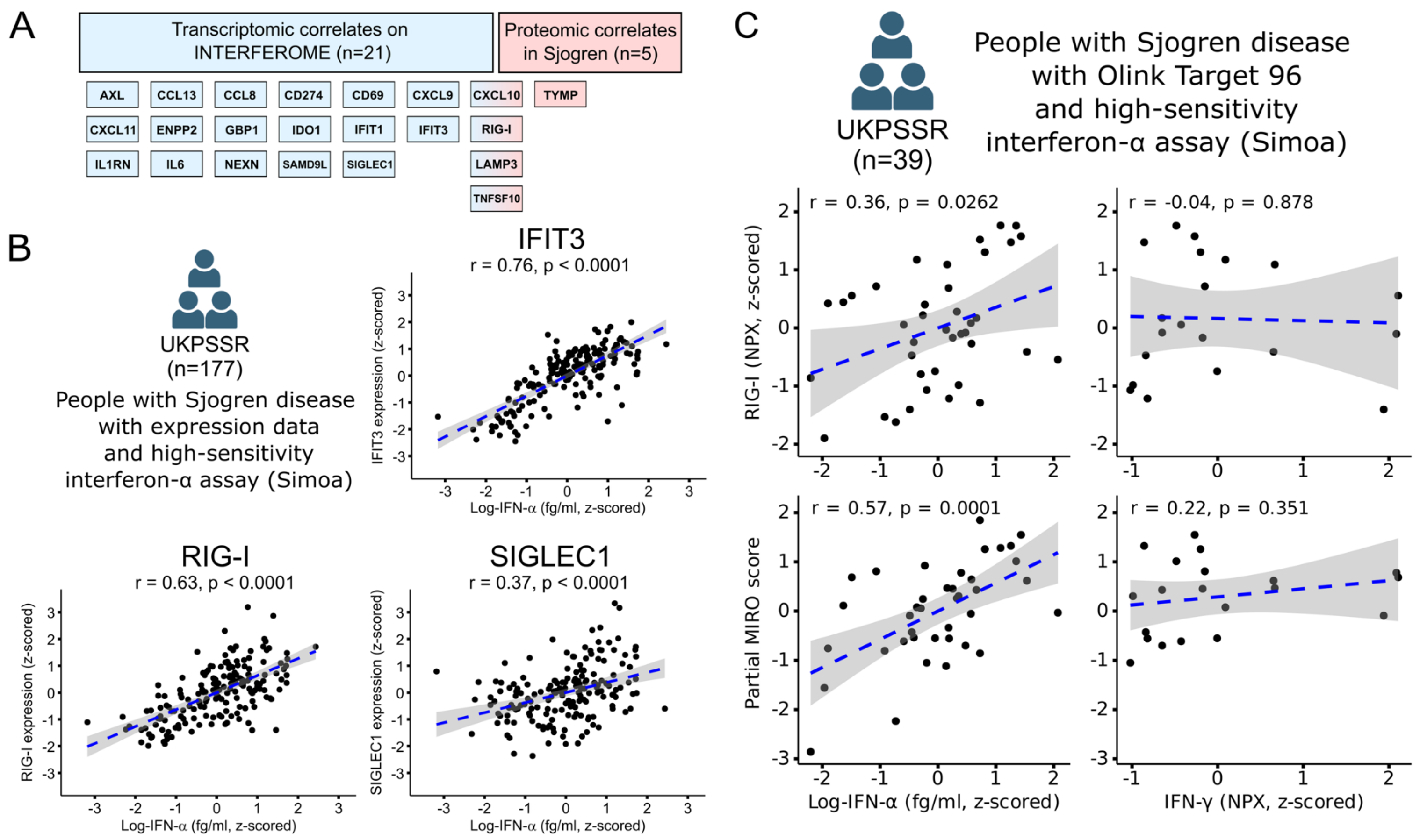

Supplementary Figure 3. Derivation and validation of the Markers of type I Interferon Response in Olink (MIRO) score.

A: Candidate correlates of type I interferons included in the penalized logistic regression.
B: Genes encoding the MIRO score are interferon-stimulated genes. Components of the MIRO score correlate significantly with interferon-α levels in UKPSSR (Pearson's correlation coefficients, two-sided tests).
C: External validation of MIRO in 39 Sjogren disease patients from UKPSSR. The partial MIRO score was calculated using RIG-I and CD163 (surrogate for SIGLEC1 with correlation between the two proteins in Sjogren disease = 0.673, $p<0.0001$). SIGLEC1 and IFIT3 were not captured in UKPSSR. Associations examined using Pearson's correlation coefficients (two-sided tests).
Partly created in BioRender. Rioux, B. (2025) https://BioRender.com/zg7bcjo/. Abbreviations: CI, confidence interval; IFN, interferon; MIRO, Markers of type I Interferon Response in Olink; NPX, normalized protein expression; UKPSSR, United Kingdom Primary Sjogren's Syndrome Registry.

Phenotype	Non-carriers, n (%)	Carriers, n (%)	aOR (95% CI)	p-value
Diseases with high interferon signature				
Sjogren disease	1,516 (0.3)	1 (0.1)	0.37 (0.04, 1.33)	0.152
Systemic sclerosis	450 (0.1)	1 (0.1)	1.26 (0.14, 4.52)	0.783
Dermatomyositis	260 (0.1)	2 (0.2)	3.62 (0.76, 10.29)	0.095
Systemic lupus erythematosus	1,303 (0.3)	3 (0.2)	0.96 (0.27, 2.36)	0.939
Any	3,229 (0.7)	7 (0.6)	0.86 (0.38, 1.65)	0.681
Diseases with moderate interferon signature				
Primary biliary cirrhosis	575 (0.1)	0 (0.0)	0.351 (0.003, 2.379)	0.371
Coeliac disease	4,108 (0.9)	5 (0.4)	0.54 (0.20, 1.12)	0.107
Vasculitis	2,160 (0.5)	6 (0.5)	1.19 (0.49, 2.37)	0.666
Vitiligo	1,160 (0.2)	1 (0.1)	0.48 (0.06, 1.71)	0.309
Rheumatoid arthritis	12,502 (2.7)	25 (2.1)	0.79 (0.52, 1.14)	0.223
Type 1 diabetes	4,962 (1.1)	18 (1.5)	1.40 (0.85, 2.16)	0.172
Any	24,398 (5.2)	52 (4.3)	0.83 (0.62, 1.08)	0.168
Diseases with lower interferon signature				
Myasthenia gravis	542 (0.1)	3 (0.2)	2.56 (0.72, 6.29)	0.130
Polymyalgia rheumatica	5,415 (1.2)	16 (1.3)	1.23 (0.72, 1.94)	0.418
Sarcoidosis	2,035 (0.4)	0 (0.0)	0.086 (0.001, 0.584)	0.004
Inflammatory bowel disease	8,490 (1.8)	19 (1.6)	0.90 (0.56, 1.37)	0.638
Hashimoto's thyroiditis	1,420 (0.3)	4 (0.3)	1.24 (0.42, 2.79)	0.657
Ankylosing spondylitis	2,176 (0.5)	8 (0.7)	1.54 (0.72, 2.83)	0.244
Psoriasis	14,926 (3.2)	35 (2.9)	0.91 (0.64, 1.26)	0.590
Pernicious anaemia	4,256 (0.9)	8 (0.7)	0.77 (0.36, 1.42)	0.433
Graves' disease	8,282 (1.8)	15 (1.2)	0.72 (0.42, 1.15)	0.177
Multiple sclerosis	2,383 (0.5)	3 (0.2)	0.59 (0.17, 1.45)	0.281
Addison's disease	757 (0.2)	1 (0.1)	0.75 (0.09, 2.66)	0.707
Any	46,647 (10.0)	107 (8.8)	0.88 (0.72, 1.07)	0.218
Neurological comorbidity of lupus				
Depression	58,537 (12.5)	141 (11.6)	0.95 (0.79, 1.12)	0.530
Epilepsy	8,347 (1.8)	19 (1.6)	0.91 (0.56, 1.38)	0.672
Ischemic stroke	16,609 (3.5)	43 (3.5)	1.03 (0.75, 1.38)	0.838
Migraine	25,607 (5.5)	66 (5.4)	1.01 (0.78, 1.29)	0.915
Psychosis	1,428 (0.3)	2 (0.2)	0.62 (0.13, 1.75)	0.416
Vascular dementia	2,073 (0.4)	4 (0.3)	0.87 (0.29, 1.95)	0.757
Any	97,572 (20.8)	235 (19.3)	0.93 (0.80, 1.07)	0.319

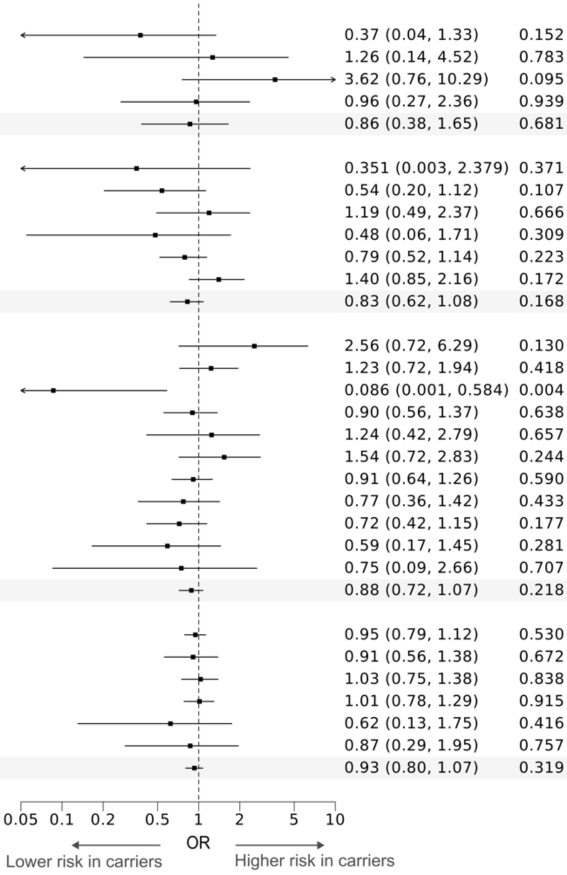

Supplementary Figure 4. Clinical associations of reported disease-causing *TREX1* variants.

aORs obtained from logistic regressions adjusted for age, sex and the 10 first genetic principal components (two-sided tests). Abbreviations: aOR, adjusted odds ratio; CI, confidence interval; MIRO, Markers of type I Interferon Response in Olink.

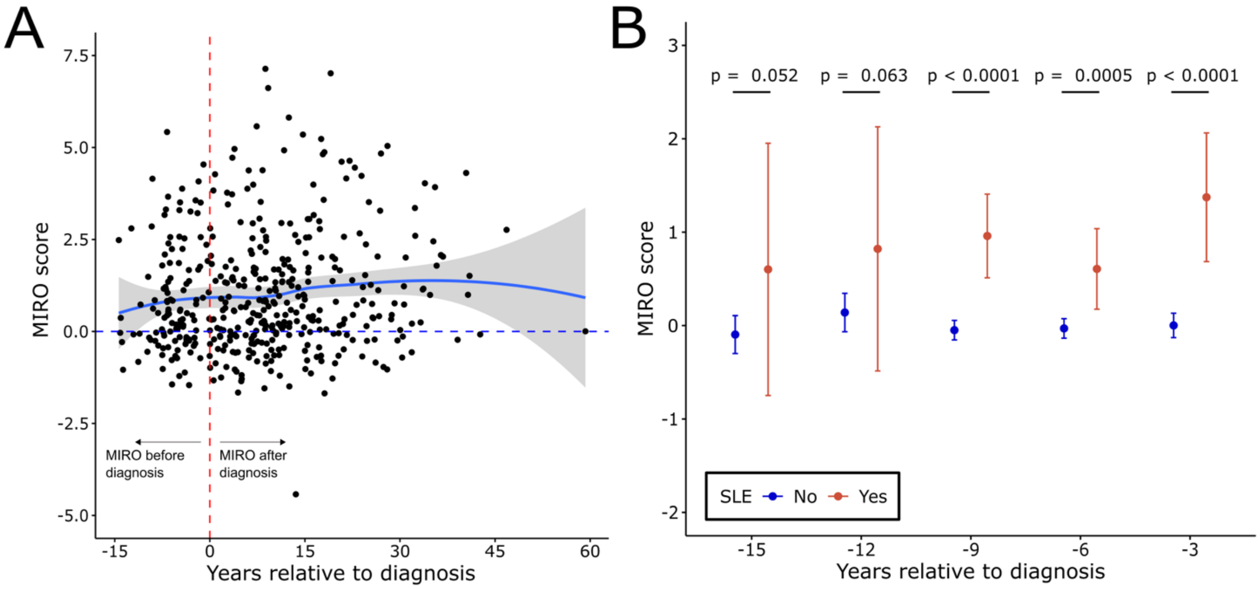

Supplementary Figure 5. Detection of oligoprotein interferon signatures before diagnosis of lupus in UK Biobank.

A: Distribution of MIRO scores relative to recorded SLE diagnosis in UK Biobank. The blue dotted line at y=0 indicates the mean MIRO score in UKB. Smooth curve produced by locally estimated scatterplot smoothing (LOESS) along with standard errors (grey).
B: Analysis of MIRO scores by 3-year epoch prior to SLE diagnosis in UK Biobank. Participants with SLE were matched 1:10 to controls selected at baseline on age and sex. Plot shows mean MIRO scores with 95% confidence intervals by group and p-values obtained through linear regressions (two-sided tests). Intervals are closed to the left and open to the right, i.e. -15: [-15, -12), -12: [-12, -9), -9: [-9, -6), -6: [-6, -3), -3: [-3, 0).
Abbreviations: MIRO, Marker of type I Interferon Response in Olink; SLE, systemic lupus erythematosus.

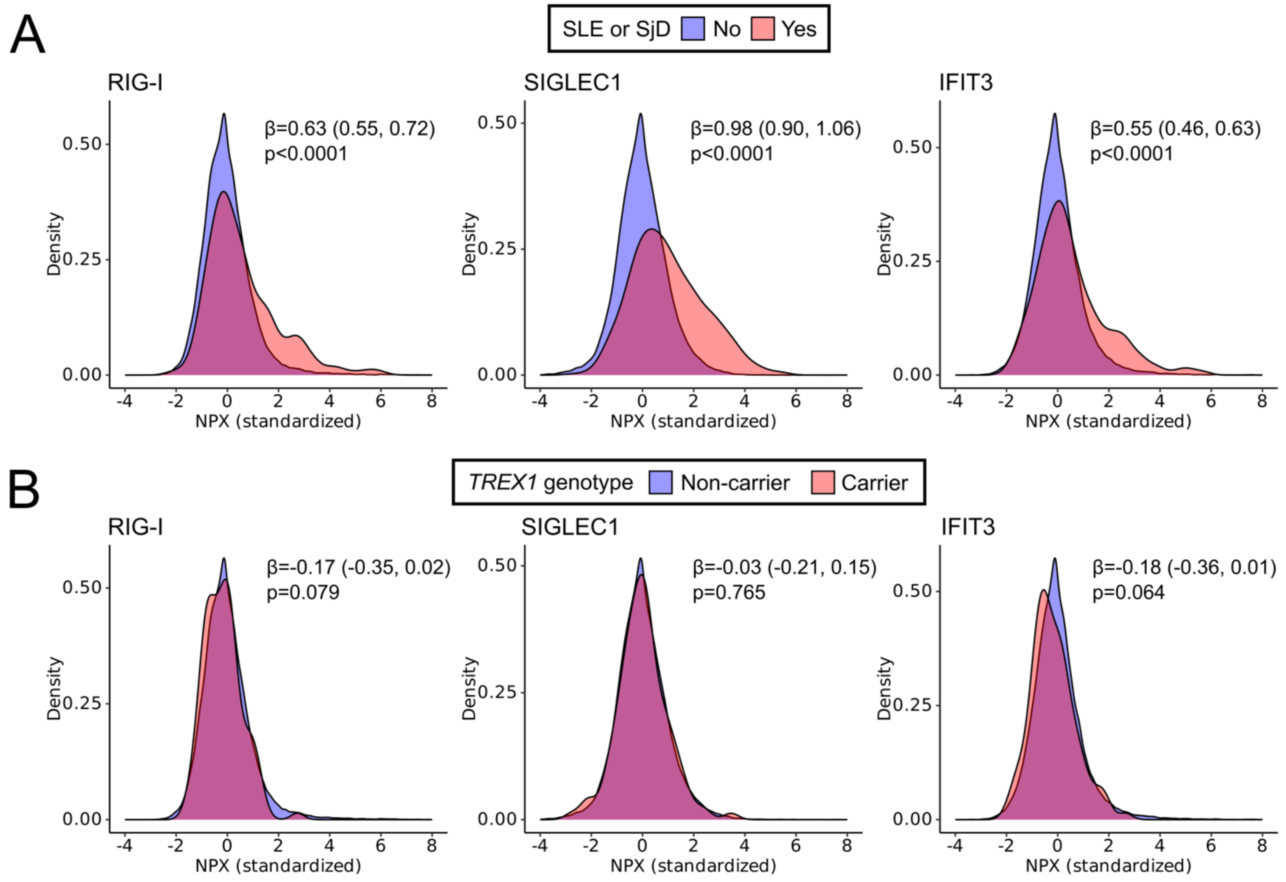

Supplementary Figure 6. Density plots of components of the MIRO score by (A) SLE/SjD status and (B) *TREX1* genotype in UK Biobank.

Beta (95% confidence interval) from linear regressions on standardized NPX values, adjusted for age and sex (and 10 first genetic principal components for *TREX1* genotype, two-sided tests).
Abbreviations: MIRO, Markers of type I Interferon Response in Olink; NPX, normalized protein expression; SjD, Sjogren disease; SLE, systemic lupus erythematosus.

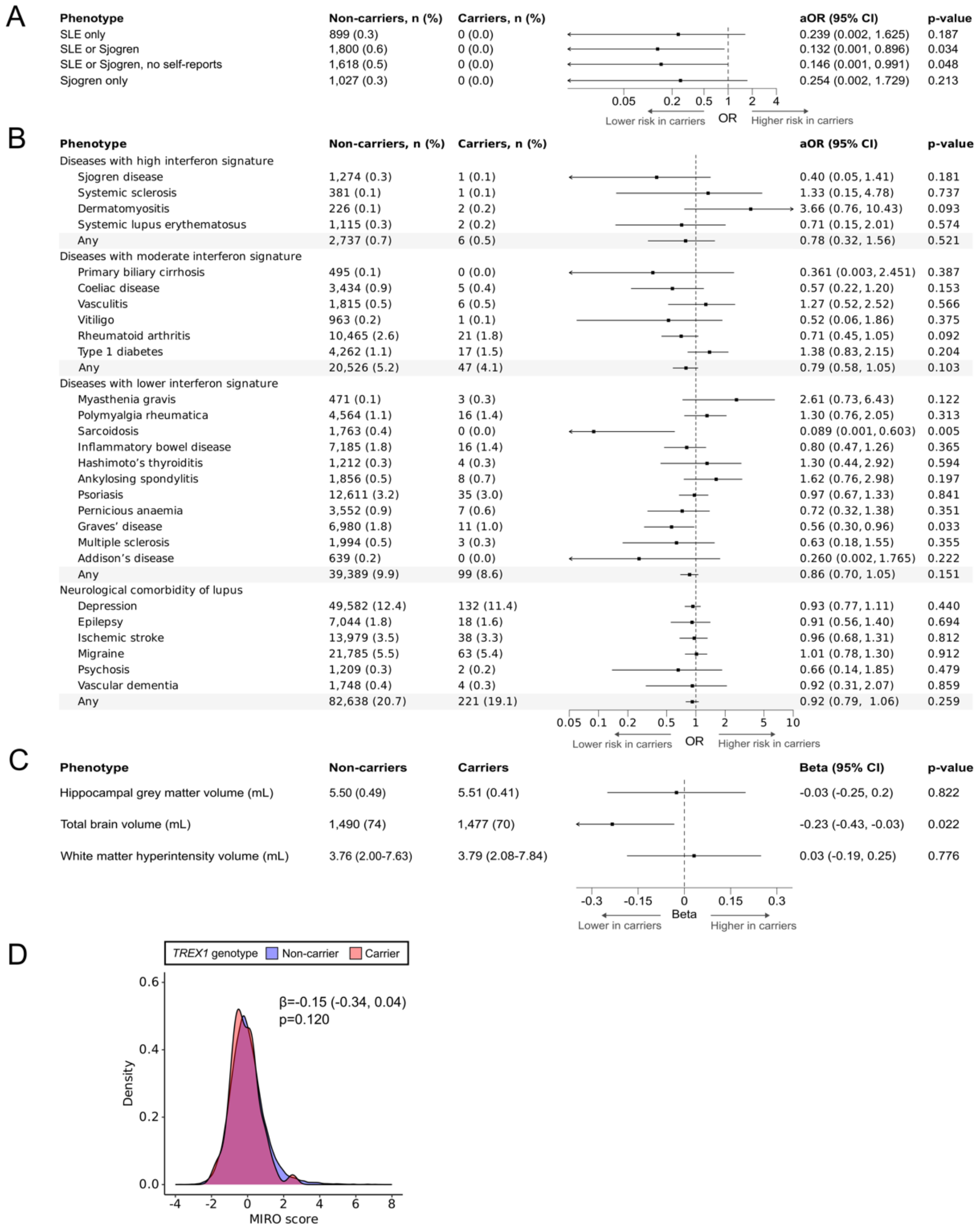

Supplementary Figure 7. Replication of analyses in genetically unrelated UK Biobank participants.

A: Clinical associations of previously reported *TREX1* lupus risk variants. Logistic regressions with Firth penalization adjusted for age, sex and the 10 first genetic principal components (two-sided tests).
B: Clinical associations of reported disease-causing *TREX1* variants. Logistic regressions with Firth penalization adjusted for age, sex and the 10 first genetic principal components (two-sided tests).
C: Neuroradiological associations of reported disease-causing *TREX1* variants. Linear regressions adjusted for age, sex, scanning centre and the 10 first genetic principal components (two-sided tests).
D: Density plot of MIRO score by *TREX1* genotype. Linear regression adjusted for age, sex and the 10 first genetic principal components (two-sided test).
Abbreviations: aOR, adjusted odds ratio; CI, confidence interval; MIRO, Marker of type I Interferon Response in Olink; SLE, systemic lupus erythematosus.

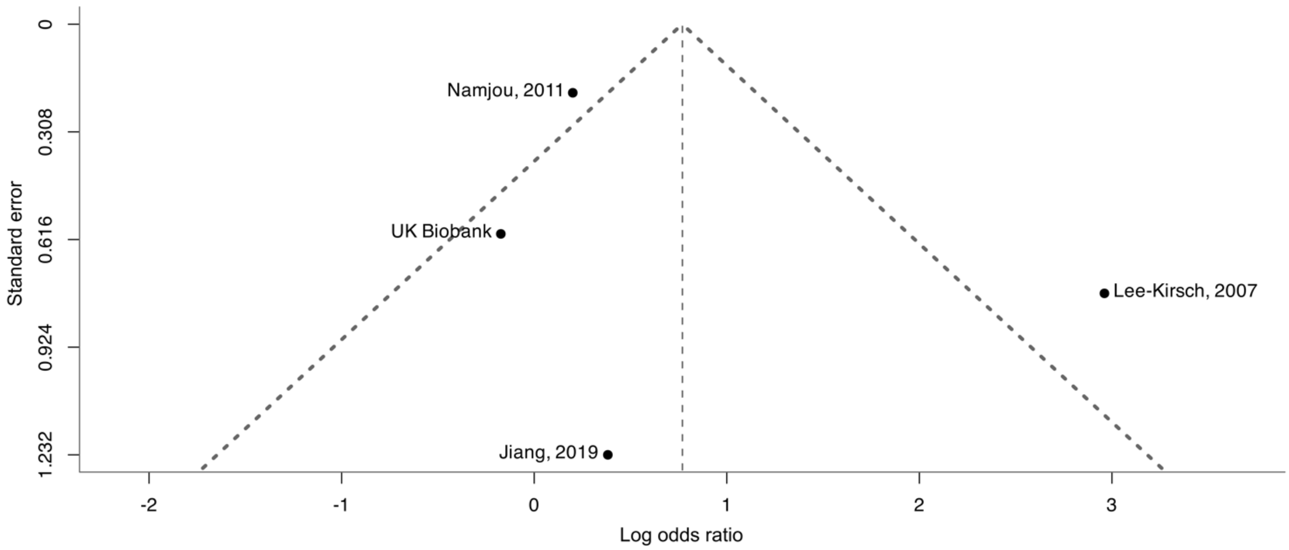

Supplementary Figure 8. Funnel plot for *TREX1*-lupus association studies included in the review.

Source data are provided as a Source Data file.

SUPPLEMENTARY TABLES

Supplementary Table 1. Demographics of participants by *TREX1* genotype.

Characteristic	Non-carriers (n=468,363)	Carriers (n=866)
**Age at cohort inception, mean (SD; range)**	54.2 (8.1; 34.3-72.0)	53.1 (8.3; 36.1-68.8)
**Female sex, n (%)**	253,919 (54.2)	444 (51.3)
**Ethnic background, n (%)**		
**White**	441,593 (94.3)	740 (85.5)
**Asian or Asian British**	9,084 (1.9)	6 (0.7)
**Black or Black British**	7,183 (1.5)	86 (9.9)
**Other**	4,154 (0.9)	16 (1.8)
**Mixed**	2,709 (0.6)	10 (1.2)
**Chinese**	1,454 (0.3)	1 (0.1)
**Missing**	2,186 (0.5)	7 (0.8)
**European ancestry, n (%)**	392,801 (83.9)	632 (73.0)

Cohort inception defined as 13 March 2006 (i.e., date of first on-site assessment). Abbreviations: SD, standard deviation.

Supplementary Table 2. Sources of diagnosis for systemic lupus erythematosus.

Source	n	%
**Death only**	0	0.0
**Death and other(s)**	27	2.1
**Primary care only**	188	14.4
**Primary care and other(s)**	286	21.9
**Hospital only**	408	31.2
**Hospital and other(s)**	240	18.4
**Self-report only**	157	12.0
**Total**	**1,306**	**100.0**

Sources were considered regardless of their chronological order of appearance, and primary sources of diagnosis were assigned based on the same arbitrary order used by UK Biobank (i.e., death registries first, followed by primary care records, hospital records, and self-reports). For example, a participant with diagnostic codes in both hospital records and death registries would have as source ‘death and other(s)’.

Supplementary Table 3. Demographics of participants by systemic lupus erythematosus status and data availability.

Characteristic	Without SLE			With SLE		
	All (n=467,923)	MIRO available (n= 34,932)	Brain MRI available (n= 43,728)	All (n=1,306)	MIRO available (n=432)	Brain MRI available (n=86)
**Age at cohort inception, mean (SD; range)**	54.2 (8.1; 34.3-72.0)	54.5 (8.2; 36.0-68.9)	52.6 (7.5; 36.1-68.8)	54.4 (8.1; 36.3-68.6)	54.5 (7.9; 36.5-68.1)	53.1 (7.1; 37.5-64.5)
**Age at diagnosis, n (%)**	NA	NA	NA			
**<20**				23 (1.8)	6 (1.4)	2 (2.3)
**[20-40)**				306 (23.4)	118 (27.3)	19 (22.1)
**[40-60)**				560 (42.9)	200 (46.3)	47 (54.7)
**≥60**				412 (31.5)	107 (24.8)	17 (19.8)
**Missing**				5 (0.4)	1 (0.2)	1 (1.2)
**Female sex, n (%)**	253,282 (54.1)	18,758 (53.7)	22,964 (52.5)	1,081 (82.8)	372 (86.1)	72 (83.7)
**Ethnic background, n (%)**						
**White**	441,186 (94.3)	32,609 (93.3)	42,330 (96.8)	1,147 (87.8)	371 (85.9)	83 (96.5)
**Asian/Asian British**	9,038 (1.9)	632 (1.8)	455 (1.0)	52 (4.0)	17 (3.9)	1 (1.2)
**Black/Black British**	7,201 (1.5)	800 (2.3)	268 (0.6)	68 (5.2)	32 (7.4)	2 (2.3)
**Other**	4,156 (0.9)	412 (1.2)	226 (0.5)	14 (1.1)	5 (1.2)	0 (0.0)
**Mixed**	2,705 (0.6)	219 (0.6)	200 (0.5)	14 (1.1)	4 (0.9)	0 (0.0)
**Chinese**	1,452 (0.3)	101 (0.3)	131 (0.3)	3 (0.2)	2 (0.5)	0 (0.0)
**Missing**	2,185 (0.5)	159 (0.5)	118 (0.3)	8 (0.6)	1 (0.2)	0 (0.0)
**European ancestry, n (%)**	392,452 (83.9)	29,045 (83.1)	37,952 (86.8)	981 (75.1)	316 (73.1)	71 (82.6)

Cohort inception defined as 13 March 2006 (i.e., date of first on-site assessment). Abbreviations: MIRO, Markers of type I Interferon Response in Olink; MRI, magnetic resonance imaging; SD, standard deviation.

Supplementary Table 4. Sources of diagnosis for systemic lupus erythematosus and Sjogren disease.

Source	n	%
**Death only**	0	0.0
**Death and other(s)**	45	1.7
**Primary care only**	285	10.8
**Primary care and other(s)**	423	16.0
**Hospital only**	1,124	42.5
**Hospital and other(s)**	498	18.8
**Self-report only**	268	10.1
**Total**	**2,643**	**100.0**

Sources were considered regardless of their chronological order of appearance, and primary sources of diagnosis were assigned based on the same arbitrary order used by UK Biobank (i.e., death registries first, followed by primary care records, hospital records, and self-reports). For example, a participant with diagnostic codes in both hospital records and death registries would have as source ‘death and other(s)’. There were 180 cases with both systemic lupus erythematosus and Sjogren disease.

Supplementary Table 5. Association of reported *TREX1* risk variants with systemic lupus erythematosus and Sjogren disease in UK Biobank using SAIGE-GENE+.

Phenotype	Variants weighted by MAF		Variants weighted by impact on enzymatic function (inverse of residual activity relative to wild type)	
	**Beta (SE)**	**p-value**	**Beta (SE)**	**p-value**
**All reported *TREX1* risk variants**				
SLE only	-0.041 (0.033)	0.289	-0.309 (0.258)	0.255
SLE or SjD	-0.028 (0.023)	0.298	-0.201 (0.180)	0.294
SLE or SjD, no self-reports	-0.026 (0.024)	0.369	-0.188 (0.190)	0.365
SjD only	-0.019 (0.030)	0.660	-0.122 (0.235)	0.683
**Excluding c.341G>A (R114H)**				
SLE only	-0.040 (0.083)	0.770	-0.807 (1.781)	0.805
SLE or SjD	-0.040 (0.058)	0.637	-0.780 (1.211)	0.681
SLE or SjD, no self-reports	-0.040 (0.061)	0.663	-0.775 (1.280)	0.707
SjD only	-0.040 (0.076)	0.745	-0.751 (1.559)	0.785

Set-based rare variant tests implemented in SAIGE-GENE+ using burden tests to generate effect sizes and SKAT-O to generate p-values (two-sided tests). Abbreviations: MAF, minor allele frequency; SE, standard error; SjD, Sjogren disease; SKAT-O, sequence kernel association test - optimal; SLE, systemic lupus erythematosus.

Supplementary Table 6. Definition of expanded clinical and radiological phenotypes.

Phenotype	Classification source (UKB fields): diagnostic codes
**Autoimmune diseases with high interferon signature (n=4)**	
Dermatomyositis	UKB first occurrence (131896, 131897):<br>**ICD v10**: M33 (dermatopolymyositis).
Sjogren disease	Mapped using codes from Conrad, 2022[1]
Systemic lupus erythematosus	Mapped using codes from Conrad, 2022[1]
Systemic sclerosis	UKB first occurrence (131898, 131899):<br>**ICD v10**: M34 (systemic sclerosis).
**Autoimmune diseases with moderate interferon signature (n=6)**	
Coeliac disease	Mapped using codes from Conrad, 2022[1]
Primary biliary cirrhosis	Mapped using codes from Conrad, 2022[1]
Rheumatoid arthritis	UKB first occurrence (131848, 131849, 131850, 131851, 131854, 131855):<br>**ICD v10**: M05 (seropositive rheumatoid arthritis), M06 (other rheumatoid arthritis), M08 (juvenile arthritis).
Type 1 diabetes	UKB first occurrence (130706, 130707):<br>**ICD v10**: E10 (type 1 diabetes mellitus).
Vasculitis	UKB first occurrence (131890, 131891, 131892, 131893):<br>**ICD v10**: M30 (polyarteritis nodosa and related conditions), M31 (other necrotizing vasculopathies).
Vitiligo	UKB first occurrence (131802, 131803):<br>**ICD v10**: L80 (vitiligo).
**Autoimmune diseases with lower interferon signature (n=11)**	
Addison's disease	Mapped using codes from Conrad, 2022[1]
Ankylosing spondylitis	Mapped using codes from Conrad, 2022[1]
Graves' disease	UKB first occurrence (130700, 130701):<br>**ICD v10**: E05 (thyrotoxicosis [hyperthyroidism]).
Hashimoto's thyroiditis	UKB first occurrence (130702, 130703):<br>**ICD v10**: E06 (thyroiditis).
Inflammatory bowel disease	UKB first occurrence (131626, 131627, 131628, 131629):<br>**ICD v10**: K50 (Crohn disease [regional enteritis]), K51 (ulcerative colitis).
Multiple sclerosis	UKB first occurrence (131042, 131043):

Phenotype	Classification source (UKB fields): diagnostic codes
	**ICD v10**: G35 (multiple sclerosis).
Myasthenia gravis	UKB first occurrence (131092, 131093):<br>**ICD v10**: G70 (myasthenia gravis and other myoneural disorders).
Pernicious anaemia	Mapped using codes from Conrad, 2022[1]
Polymyalgia rheumatica	Mapped using codes from Conrad, 2022[1]
Psoriasis	UKB first occurrence (131742, 131743):<br>**ICD v10**: L40 (psoriasis).
Sarcoidosis	UKB first occurrence (130686, 130687):<br>**ICD v10**: D86 (sarcoidosis).
**Neurological comorbidities of lupus (n=6)**	
Depression	UKB first occurrence (130894, 130895):<br>**ICD v10**: F32 (depressive episode).
Epilepsy	UKB first occurrence (131048-131051):<br>**ICD v10**: G40 (epilepsy), G41 (status epilepticus).
Ischemic stroke	UKB adjudication algorithm (42008, 42009):<br>**ICD v9**: 434.X (occlusion of cerebral arteries), 434.0 (cerebral thrombosis), 434.1 (cerebral embolism), 434.9 (cerebral artery occlusion, unspecified), 436.X (acute, but ill-defined, cerebrovascular disease);<br>**ICD v10**: I63 (cerebral infarction), I63.0 (cerebral infarction due to thrombosis of precerebral arteries), I63.1 (cerebral infarction due to embolism of precerebral arteries), I63.2 (cerebral infarction due to unspecified occlusion or stenosis of precerebral arteries), I63.3 (cerebral infarction due to thrombosis of cerebral arteries), I63.4 (cerebral infarction due to embolism of cerebral arteries), I63.5 (cerebral infarction due to unspecified occlusion or stenosis of cerebral arteries), I63.6 (cerebral infarction due to cerebral venous thrombosis, nonpyogenic), I63.8 (other cerebral infarction), I63.9 (cerebral infarction, unspecified), I64.X (stroke, not specified as haemorrhage or infarction);<br>**Self-report**: 20002/1583 (ischemic stroke).<br>UKB first occurrence (131366-131369):<br>**ICD v10**: I63 (cerebral infarction), I64 (stroke, not specified as haemorrhage or infarction).
Migraine	UKB first occurrence (131052, 131053):<br>**ICD v10**: G43 (migraine).
Psychosis	UKB first occurrence (130878-130881, 130886-130889):<br>**ICD v10**: F22 (persistent delusional disorders), F23 (acute and transient psychotic disorders), F28 (other nonorganic psychotic disorders), F29 (unspecified nonorganic psychosis).

Phenotype	Classification source (UKB fields): diagnostic codes
Vascular dementia	UKB adjudication algorithm (42022, 42023):<br>**ICD v9**: 290.4 (arteriosclerotic dementia);<br>**ICD v10**: F01 (vascular dementia), F01.0 (vascular dementia of acute onset), F01.1 (multi-infarct dementia), F01.2 (subcortical vascular dementia), F01.3 (mixed cortical and sub-cortical vascular dementia), F01.8 (other vascular dementia), F01.9 (vascular dementia, unspecified), I67.3 (Binswanger's disease).<br>UKB first occurrence (130838, 130839):<br>**ICD v10**: F01 (vascular dementia).
**Control conditions (n=7)**	
Chronic kidney disease	UKB first occurrence (131290-131293, 132032-132035):<br>**ICD v10**: I12 (hypertensive renal disease), I13 (hypertensive heart and renal disease), N18 (chronic kidney disease), N19 (unspecified kidney failure).
Chronic viral hepatitis	UKB first occurrence (130200, 130201):<br>**ICD v10**: B18 (chronic viral hepatitis).
Haemorrhoids	UKB first occurrence (131404, 131405, 131650, 131651):<br>**ICD v10**: I84 (haemorrhoids), K64 (haemorrhoids and perianal venous thrombosis).
Human immunodeficiency virus infection	UKB first occurrence (130204-130213):<br>**ICD v10**: B20 (human immunodeficiency virus [HIV] disease resulting in infectious and parasitic diseases), B21 (human immunodeficiency virus [HIV] disease resulting in malignant neoplasms), B22 (human immunodeficiency virus [HIV] disease resulting in other specified diseases), B23 (human immunodeficiency virus [HIV] disease resulting in other conditions), B24 (unspecified human immunodeficiency virus [HIV] disease)
Interferon medication	Treatment/medication name reported at baseline (20003):<br>Pattern matching search (grepl) for interferon beta, interferon beta-1a, rebif, avonex, interferon beta-1b, betaferon, extavia, peginterferon alfa, peginterferon alpha, interferon alfa, interferon alpha, pegasys, introna, pegintron, roferon-a. Participants with multiple sclerosis reporting treatment with "interferons" were considered to receive interferon beta.
Liver disease	UKB first occurrence (130200, 130201, 131658, 131659, 131662, 131663, 131664, 131665, 131670, 131671):<br>**ICD v10**: B18 (chronic viral hepatitis), K70 (alcoholic liver disease), K72 (hepatic failure, not elsewhere classified), K73 (chronic hepatitis, not elsewhere classified), K76 (other diseases of liver).
Organ/tissue transplant	Hospital records:<br>**ICD v9**: V42 (organ or tissue replaced by transplant);

Phenotype	Classification source (UKB fields): diagnostic codes
	**ICD v10**: Z94 (transplanted organ and tissue status)
**Neuroradiological phenotypes (n=3)**	
Hippocampal grey matter volume	Volume of grey matter in hippocampus, average of right and left measurements (25886, 25887), mm^3
Total brain volume	Volume of brain (grey and white matter) normalized for head size (25009), mm^3
White matter hyperintensity volume	Total volume of white matter hyperintensities (25781), mm^3

Abbreviations: ICD, International Classification of Diseases; MIRO, Marker of type I Interferon Response in Olink; UKB, UK Biobank.

Supplementary Table 7. Mapping of phenotypes that were not defined from UK Biobank first occurrence fields.

Classification	Code	Definition
**Addison's disease**		
ICD v10	E27.1	Primary adrenocortical insufficiency
ICD v10	E27.2	Addisonian crisis
ICD v9	2554	Glucocorticoid deficiency
Read v2	D010.	Addison's anaemia
Read v2	C1540	Addisonian crisis
Read v2	C1541	Addison's disease
Read v2	C1546	Addisonian crisis
Read v2	Cyu49	[X]Other+unspecified primary adrenocortical insufficiency
Read v2	F3950	Myopathy due to Addison's disease
Read v3	D010.	Addison's anaemia
Read v3	C1540	Addisonian crisis
Read v3	C1541	Addison's disease
Read v3	C1546	Addisonian crisis
Read v3	Cyu49	[X]Other+unspecified primary adrenocortical insufficiency
Read v3	F3950	Myopathy due to Addison's disease
Read v3	X40Md	Addisonian crisis
Read v3	X40Mf	Autoimmune Addison's disease
Self-report	1234	Adrenocortical insufficiency/addison's disease
**Ankylosing spondylitis**		
ICD v10	M45	Ankylosing spondylitis
ICD v10	M45.0	Ankylosing spondylitis - Multiple sites in spine
ICD v10	M45.1	Ankylosing spondylitis - Occipito-atlanto-axial region
ICD v10	M45.2	Ankylosing spondylitis - Cervical region
ICD v10	M45.3	Ankylosing spondylitis - Cervicothoracic region
ICD v10	M45.4	Ankylosing spondylitis - Thoracic region
ICD v10	M45.5	Ankylosing spondylitis - Thoracolumbar region
ICD v10	M45.6	Ankylosing spondylitis - Lumbar region
ICD v10	M45.7	Ankylosing spondylitis - Lumbosacral region
ICD v10	M45.8	Ankylosing spondylitis - Sacral and sacrococcygeal region
ICD v10	M45.9	Ankylosing spondylitis - Site unspecified
ICD v9	7200	Ankylosing spondylitis
Read v2	N100.	Ankylosing spondylitis
Read v2	2377	O/E - ankyl.spondyl.chest def.
Read v2	N0450	Juvenile ankylosing spondylitis
Read v2	43cG.	Human leucocyte antigen B27 positive
Read v2	N11F.	Axial spondyloarthritis
Read v3	N100.	Ankylosing spondylitis
Read v3	2377	O/E - ankyl.spondyl.chest def.
Read v3	N0450	Juvenile ankylosing spondylitis
Read v3	43cG.	Human leucocyte antigen B27 positive
Read v3	N11F.	Axial spondyloarthritis
Read v3	X7024	AS with multisystem involvement

Self-report	1313	Ankylosing spondylitis
**Coeliac disease**		
ICD v10	K90.0	Coeliac disease
ICD v9	5790	Celiac disease
Read v2	J690.	Coeliac disease
Read v2	J690z	Coeliac disease NOS
Read v2	J6900	Congenital coeliac disease
Read v2	J6901	Acquired coeliac disease
Read v2	68W4.	Coeliac disease autoantibody profile positive
Read v3	J690.	Coeliac disease
Read v3	J690z	Coeliac disease NOS
Read v3	J6900	Congenital coeliac disease
Read v3	J6901	Acquired coeliac disease
Read v3	68W4.	Coeliac disease autoantibody profile positive
Read v3	X3037	Adult coeliac disease
Read v3	XE0bK	Coeliac disease
Read v3	XE0bL	Childhood gluten enteropathy
Self-report	1456	Malabsorption/coeliac disease
**Pernicious anaemia**		
ICD v10	K29.4	Chronic atrophic gastritis
ICD v10	D51.0	Vitamin B12 deficiency anaemia due to intrinsic factor deficiency
ICD v9	2810	Pernicious anemia
Read v2	D010.	Pernicious anaemia
Read v2	D011.	Vitamin B12 deficiency anaemia
Read v2	D011X	Vitamin B12 deficiency anaemia, unspecified
Read v2	D011z	Other vitamin B12 deficiency anaemia NOS
Read v2	D0110	Vitamin B12 deficiency anaemia due to dietary causes
Read v2	D0130	Combined B12 and folate deficiency anaemia
Read v2	Dyu02	[X]Other vitamin B12 deficiency anaemias
Read v2	Dyu01	[X]Other dietary vitamin B12 deficiency anaemia
Read v2	Dyu06	[X]Vitamin B12 deficiency anaemia, unspecified
Read v2	J1510	Chronic atrophic gastritis
Read v2	F11x6	Cerebral degeneration due to vitamin B12 deficiency
Read v2	F3815	Myasthenic syndrome due to pernicious anaemia
Read v3	D010.	Pernicious anaemia
Read v3	D011.	Vitamin B12 deficiency anaemia
Read v3	D011X	Vitamin B12 deficiency anaemia, unspecified
Read v3	D011z	Other vitamin B12 deficiency anaemia NOS
Read v3	D0110	Vitamin B12 deficiency anaemia due to dietary causes
Read v3	D0130	Combined B12 and folate deficiency anaemia
Read v3	Dyu02	[X]Other vitamin B12 deficiency anaemias
Read v3	Dyu01	[X]Other dietary vitamin B12 deficiency anaemia
Read v3	Dyu06	[X]Vitamin B12 deficiency anaemia, unspecified
Read v3	J1510	Chronic atrophic gastritis
Read v3	F11x6	Cerebral degeneration due to vitamin B12 deficiency
Read v3	F3815	Myasthenic syndrome due to pernicious anaemia
Read v3	XE13g	B12-defic megaloblastic anaem

Read v3	XE2ro	Pernicious anaemia
Read v3	Xa9Aw	B12-defic megaloblastic anaem
Self-report	1331	Pernicious anaemia
**Polymyalgia rheumatica**		
ICD v10	M31.5	Giant cell arteritis with polymyalgia rheumatica
ICD v10	M35.3	Polymyalgia rheumatica
ICD v9	725	Polymyalgia rheumatica
Read v2	N20..	Polymyalgia rheumatica
Read v2	N200.	Giant cell arteritis with polymyalgia rheumatica
Read v3	N20..	Polymyalgia rheumatica
Read v3	N200.	Giant cell arteritis with polymyalgia rheumatica
Self-report	1377	Polymyalgia rheumatica
**Primary biliary cirrhosis**		
ICD v10	K74.3	Primary biliary cirrhosis
ICD v10	K74.5	Biliary cirrhosis, unspecified
ICD v9	5716	Biliary cirrhosis
Read v2	J616.	Biliary cirrhosis
Read v2	J6160	Primary biliary cirrhosis
Read v2	J6162	Biliary cirrhosis of children
Read v2	J616z	Biliary cirrhosis NOS
Read v3	J616.	Biliary cirrhosis
Read v3	J6160	Primary biliary cirrhosis
Read v3	J6162	Biliary cirrhosis of children
Read v3	J616z	Biliary cirrhosis NOS
Read v3	.I726	Biliary cirrhosis
Read v3	XE0dJ	Biliary cirrhosis
Self-report	1506	Primary biliary cirrhosis
**Sjogren disease**		
ICD v10	M35.0	Sicca syndrome [Sjögren]
ICD v9	7102	Sicca syndrome
Read v2	F3967	Myopathy due to Sjogren's disease
Read v2	H57y3	Lung disease with Sjogren's disease
Read v2	N002.	Sicca (Sjogren's) syndrome
Read v3	H57y3	Lung disease with Sjogren's disease
Read v3	N002.	Sicca (Sjogren's) syndrome
Read v3	X705A	Primary Sjogren's syndrome
Read v3	X705B	Primary Sjogren's syndrome with organ/system involvement
Read v3	X705C	Primary Sjogren's syndrome with multisystem involvement
Read v3	X705D	Secondary Sjogren's syndrome
Read v3	X705E	Secondary Sjogren's syndrome with organ/system involvement
Read v3	X705F	Secondary Sjogren's syndrome with multisystem involvement
Read v3	XE1Ge	Sjogren's disease
Self-report	1382	Sjögren's syndrome/sicca syndrome
**Systemic lupus erythematosus**		
ICD v10	L93	Lupus erythematosus
ICD v10	L93.0	Discoid lupus erythematosus
ICD v10	L93.1	Subacute cutaneous lupus erythematosus

ICD v10	L93.2	Other local lupus erythematosus
ICD v10	M32	Systemic lupus erythematosus
ICD v10	M32.1	Systemic lupus erythematosus with organ or system involvement
ICD v10	M32.8	Other forms of systemic lupus erythematosus
ICD v10	M32.9	Systemic lupus erythematosus, unspecified
ICD v9	6954	Lupus erythematosus
ICD v9	7100	Systemic lupus erythematosus
Read v2	F3710	Polyneuropathy in disseminated lupus erythematosus
Read v2	F3961	Myopathy due to disseminated lupus erythematosus
Read v2	F4D33	Eyelid discoid lupus erythematosus
Read v2	H57y4	Lung disease with systemic lupus erythematosus
Read v2	K01x4	Nephrotic syndrome in systemic lupus erythematosus/lupus nephritis
Read v2	K0B40	Renal tubulo-interstitial disorder in SLE
Read v2	M154.	Lupus erythematosus
Read v2	M1540	Lupus erythematosus chronicus
Read v2	M1541	Discoid lupus erythematosus
Read v2	M1542	Lupus erythematosus migrans
Read v2	M1543	Lupus erythematosus nodularis
Read v2	M1544	Lupus erythematosus profundus
Read v2	M1545	Lupus erythematosus tumidus
Read v2	M1546	Lupus erythematosus unguium mutilans
Read v2	M1547	Subacute cutaneous lupus erythematosus
Read v2	M154z	Lupus erythematosus NOS
Read v2	Myu78	[X]Other local lupus erythematosus
Read v2	N000.	Systemic lupus erythematosus
Read v2	N0000	Disseminated lupus erythematosus
Read v2	N0001	Libman-Sacks disease
Read v2	N0003	Systemic lupus erythematosus with organ or system involvement
Read v2	N0004	Systemic lupus erythematosus with pericarditis
Read v2	N0006	Cerebral lupus
Read v2	N000z	Systemic lupus erythematosus NOS
Read v2	Nyu43	[X]Other forms of systemic lupus erythematosus
Read v3	F3710	Polyneuropathy in disseminated lupus erythematosus
Read v3	F3961	Myopathy due to disseminated lupus erythematosus
Read v3	F4D33	Eyelid discoid lupus erythematosus
Read v3	H57y4	Lung disease with systemic lupus erythematosus
Read v3	K01x4	Nephrotic syndrome in systemic lupus erythematosus/lupus nephritis
Read v3	K0B40	Renal tubulo-interstitial disorder in SLE
Read v3	M154.	Lupus erythematosus
Read v3	M1540	Lupus erythematosus chronicus
Read v3	M1541	Discoid lupus erythematosus
Read v3	M1542	Lupus erythematosus migrans
Read v3	M1543	Lupus erythematosus nodularis
Read v3	M1544	Lupus erythematosus profundus

Read v3	M1545	Lupus erythematosus tumidus
Read v3	M1546	Lupus erythematosus unguium mutilans
Read v3	M1547	Subacute cutaneous lupus erythematosus
Read v3	M154z	Lupus erythematosus NOS
Read v3	Myu78	[X]Other local lupus erythematosus
Read v3	N000.	Systemic lupus erythematosus
Read v3	N0000	Systemic lupus erythematosus
Read v3	N0001	Libman-Sacks disease
Read v3	N0003	Systemic lupus erythematosus with organ or system involvement
Read v3	N0004	Systemic lupus erythematosus with pericarditis
Read v3	N0006	Cerebral lupus
Read v3	N000z	Systemic lupus erythematosus NOS
Read v3	Nyu43	[X]Other forms of systemic lupus erythematosus
Read v3	X0046	Chorea in systemic lupus erythematosus
Read v3	X00Dx	Systemic lupus erythematosus encephalitis
Read v3	X50Ew	Lupus erythematosus and erythema multiforme-like syndrome
Read v3	X50Ex	Chronic discoid lupus erythematosus
Read v3	X50Ez	Chilblain lupus erythematosus
Read v3	X704W	Limited lupus erythematosus
Read v3	X704X	Systemic lupus erythematosus with organ/system involvement
Read v3	X704a	Lupus panniculitis
Read v3	X704b	Bullous systemic lupus erythematosus
Read v3	X704c	Systemic lupus erythematosus with multisystem involvement
Read v3	X704d	Cutaneous lupus erythematosus
Read v3	X704h	Subacute cutaneous lupus erythematosus
Read v3	X705w	Lupus vasculitis
Read v3	XaBE1	Renal tubulo-interstitial disorder in systemic lupus erythematosus
Read v3	XaC1J	Systemic lupus erythematosus with pericarditis
Self-report	1381	Systemic lupus erythematosus/SLE

Abbreviations: ICD, International Classification of Diseases.

Supplementary Table 8. Description of the 84 disease-causing *TREX1* variants included.

HGVS coding DNA; protein	rsid	Position; Ref:Alt	Type	Pathogenicity	Consequence	CADD (scaled)	Allele frequency (%)	
							gnomAD	1000GP
c.1A>G; p.Met1Val	rs761165865	48466656; A:G	SNV	Conflicting: LP (1); VUS (2)	Start lost	24.2	0.0016	.
c.5del; p.Gly2AlafsTer63	NA	48466657; TG:T	DEL	P	Frameshift	21.6	.	.
c.18dup; p.Pro7AlafsTer95	NA	48466672; T:TG	DUP	P	Frameshift	11.4	.	.
c.38C>A; p.Thr13Asn	NA	48466693; C:A	SNV	LP	Missense	24.8	.	.
c.52G>A; p.Asp18Asn	rs121908117	48466707; G:A	SNV	P	Missense	28.7	.	.
c.52G>C; p.Asp18His	NA	48466707; G:C	SNV	NA	Missense	28.2	.	.
c.58dup; p.Glu20GlyfsTer82	rs78300695	48466711; T:TG	DUP	P	Frameshift	28.9	0.0127	.
c.79_96del; p.Ser27_Thr32del	NA	48466733; CTCCCAGCCCA AGGTCACG:C	DEL	LP	Inframe deletion	19.9	.	.
c.95C>G; p.Thr32Arg	rs755138065	48466750; C:G	SNV	LP	Missense	26.4	0.0004	.
c.123_125dup; p.Cys42Ter	rs1354160835	48466776; A:AGAT	DUP	P/LP	Stop gained	33.0	.	.
c.137dup; p.Ser46ArgfsTer56	NA	48466791; A:AG	DUP	P/LP	Frameshift	24.0	.	.
c.143_144del; p.Pro48HisfsTer53	rs748914604	48466792; GCC:G	DEL	LP	Frameshift	21.8	.	.
c.144del; p.Thr49ProfsTer16	rs748914604	48466792; GC:G	DEL	P/LP	Frameshift	21.5	0.0004	.

HGVS coding DNA; protein	rsid	Position; Ref:Alt	Type	Pathogenicity	Consequence	CADD (scaled)	Allele frequency (%)	
							gnomAD	1000GP
c.144dup; p.Thr49HisfsTer53	rs748914604	48466792; G:GC	DUP	P/LP	Frameshift	23.0	0.0076	.
c.150_151del; p.Gln51GlyfsTer50	rs770607110	48466801; CCT:C	DEL	P	Frameshift	17.6	0.0004	.
c.152_153del; p.Gln51ArgfsTer50	rs773808155	48466806; CAG:C	DEL	P	Frameshift	19.5	0.0004	.
c.153_166del; p.Gln51HisfsTer46	NA	48466805; TCAGGGGC CACCTCC:T	DEL	P	Frameshift	22.9	.	.
c.182C>A; p.Pro61Gln	rs777034646	48466837; C:A	SNV	Conflicting: LP (1); VUS (3)	Missense	23.6	.	.
c.193_195dup; p.Asp65dup	NA	48466847; A:AGAC	DUP	LP	Inframe insertion	19.5	.	.
c.197A>G; p.Lys66Arg	rs188508043	48466852; A:G	SNV	VUS	Missense	25.7	0.0064	0.0200
c.212_213del; p.Val71GlyfsTer30	rs74689946	48466860; CTG:C	DEL	P	Frameshift	27.2	0.0012	.
c.212_213dup; p.Ala72TrpfsTer17	rs74689946	48466860; C:CTG	DUP	P	Frameshift	27.1	0.0012	.
c.218C>T; p.Pro73Leu	rs755919767	48466873; C:T	SNV	Conflicting: LP (1); VUS (5)	Missense	25.2	0.0024	.
c.236_243dup; p.Ser82LeufsTer9	rs1331920811	48466881; G:GCCTGCAGC	DUP	P/LP	Frameshift	24.6	0.0008	.
c.237_243del; p.Ala81ArgfsTer5	NA	48466889; CCCTGCAG:C	DEL	NA	Frameshift	29.7	.	.
c.240dup; p.Ala81SerfsTer21	NA	48466894; C:CA	DUP	P	Frameshift	22.6	.	.
c.243_246del; p.Ser82ArgfsTer5	rs2040335386	48466893; GCAGC:G	DEL	P	Frameshift	29.9	.	.

HGVS coding DNA; protein	rsid	Position; Ref:Alt	Type	Pathogenicity	Consequence	CADD (scaled)	Allele frequency (%)	
							gnomAD	1000GP
c.262_263insAG; p.Ser88LysfsTer23	NA	48466917; A:AAG	INS	NA	Frameshift	31.0	.	.
c.275T>A; p.Leu92Gln	NA	48466930; T:A	SNV	NA	Missense	27.1	.	.
c.290G>A; p.Arg97His	rs200773268	48466945; G:A	SNV	Conflicting: P (2); LP (2); VUS (1)	Missense	27.8	0.0060	0.0200
c.294dup; p.Cys99MetfsTer3	rs760594164	48466947; C:CA	DUP	P	Frameshift	24.9	0.0072	.
c.296_299dup; p.Phe100LeufsTer3	rs763966000	48466949; A:ATGTT	DUP	P	Frameshift	9.5	0.0008	.
c.340C>T; p.Arg114Cys	rs760838030	48466995; C:T	SNV	Conflicting: LP (3); VUS (1)	Missense	28.5	0.0008	.
c.341G>A; p.Arg114His	rs72556554	48466996; G:A	SNV	P/LP	Missense	29.3	0.0208	.
c.357G>A; p.Trp119Ter	rs747399274	48467012; G:A	SNV	P	Stop gained	37.0	.	.
c.365T>C; p.Val122Ala	rs79993407	48467020; T:C	SNV	NA	Missense	27.6	.	.
c.366_368dup; p.Ala123dup	rs77371662	48467020; T:TGGC	DUP	P	Inframe insertion	20.4	.	.
c.370C>G; p.His124Asp	rs1131691368	48467025; C:G	SNV	LP	Missense	26.5	.	.
c.374A>G; p.Asn125Ser	NA	48467029; A:G	SNV	LP	Missense	25.5	.	.
c.375dup; p.Gly126TrpfsTer2	rs1575292873	48467029; A:AT	DUP	P	Frameshift	31.0	.	.
c.383G>A; p.Arg128His	rs751124579	48467038; G:A	SNV	VUS	Missense	22.8	0.0008	.

HGVS coding DNA; protein	rsid	Position; Ref:Alt	Type	Pathogenicity	Consequence	CADD (scaled)	Allele frequency (%)	
							gnomAD	1000GP
c.388G>C; p.Asp130His	rs781051771	48467043; G:C	SNV	LP	Missense	28.7	.	.
c.393_408dup; p.Glu137ProfsTer24	rs74876396	48467047; T:TCCCCCT GCTCCAAGCA	DUP	LP	Frameshift	29.3	.	.
c.397del; p.Leu133CysfsTer27	rs78762691	48467047; TC:T	DEL	LP	Frameshift	25.9	.	.
c.399dup; p.Leu134AlafsTer22	NA	48467053; T:TG	DUP	P	Frameshift	32.0	.	.
c.403C>T; p.Gln135Ter	rs746384382|	48467058; C:T	SNV	P	Stop gained	36.0	.	.
c.415_416del; p.Ala139TyrfsTer16	NA	48467069; GGC:G	DEL	P	Frameshift	28.1	.	.
c.416del; p.Ala139ValfsTer21	rs763229085	48467070; GC:G	DEL	P	Frameshift	26.4	0.0028	.
c.467del; p.Ile156ThrfsTer4	NA	48467121; AT:A	DEL	LP	Frameshift	25.4	.	.
c.470del; p.Thr157MetfsTer3	NA	48467124; AC:A	DEL	NA	Frameshift	22.6	.	.
c.473C>T; p.Ala158Val	rs762011967	48467128; C:T	SNV	VUS	Missense	23.8	0.0044	.
c.485T>C; p.Leu162Pro	NA	48467140; T:C	SNV	NA	Missense	24.8	.	.
c.490C>T; p.Arg164Ter	rs78218009	48467145; C:T	SNV	P/LP	Stop gained	34.0	.	.
c.500del; p.Ser167ThrfsTer13	rs76642637	48467154; AG:A	DEL	P	Frameshift	21.1	0.0004	.
c.508G>T; p.Glu170Ter	rs768724007	48467163; G:T	SNV	P	Stop gained	36.0	.	.

HGVS coding DNA; protein	rsid	Position; Ref:Alt	Type	Pathogenicity	Consequence	CADD (scaled)	Allele frequency (%)	
							gnomAD	1000GP
c.513del; p.His171GlnfsTer9	rs2040356109	48467167; AC:A	DEL	P	Frameshift	6.9	.	.
c.541_544dup; p.Ile182LysfsTer18	NA	48467195; C:CAGCA	DUP	LP	Frameshift	26.1	.	.
c.553C>T; p.Arg185Cys	rs145721839	48467208; C:T	SNV	VUS	Missense	26.0	0.0020	0.0200
c.581del; p.Ser194CysfsTer83	rs1575293518	48467235; TC:T	DEL	P	Frameshift	24.7	.	.
c.585C>G; p.His195Gln	NA	48467240; C:G	SNV	NA	Missense	22.1	.	.
c.592G>A; p.Glu198Lys	rs1416519719	48467247; G:A	SNV	P	Missense	25.8	0.0004	.
c.598G>C; p.Asp200His	NA	48467253; G:C	SNV	P	Missense	25.1	.	.
c.598G>A; p.Asp200Asn	rs78846775	48467253; G:A	SNV	P	Missense	25.6	.	.
c.599_601dup; p.Asp200dup	rs74556809	48467251; G:GTGA	DUP	LP	Inframe insertion	17.0	0.0008	.
c.599_602dup; p.Leu202CysfsTer40	NA	48467253; G:GATGT	DUP	LP	Frameshift	28.5	.	.
c.602T>A; p.Val201Asp	rs78408272	48467257; T:A	SNV	P	Missense	25.4	.	.
c.609_662dup; p.Leu204_Ala221dup	rs78379807	48467261; G:GGCCCTG CTCAGCATC TGTCAGTGG AGACCACAG GCCCTGCTGC GGTGGGTGGAT	DUP	Conflicting: P (1); VUS (1)	Inframe insertion	8.3	.	.
c.621_622del; p.Ile207MetfsTer33	rs781199890	48467274; ATC:A	DEL	P/LP	Frameshift	32.0	0.0012	.

HGVS coding DNA; protein	rsid	Position; Ref:Alt	Type	Pathogenicity	Consequence	CADD (scaled)	Allele frequency (%)	
							gnomAD	1000GP
c.625_628dup; p.Trp210SerfsTer32	rs78948846	48467277; T:TGTCA	DUP	NA	Frameshift	32.0	0.0008	.
c.629G>A; p.Trp210Ter	rs184953805	48467284; G:A	SNV	P	Stop gained	39.0	.	.
c.635del; p.Pro212HisfsTer65	rs756664985	48467288; AC:A	DEL	P/LP	Frameshift	28.9	0.0004	.
c.659A>G; p.Asp220Gly	NA	48467314; A:G	SNV	LP	Missense	27.1	.	.
c.667G>A; p.Ala223Thr	rs766785968	48467322; G:A	SNV	Conflicting: LP (2); VUS (2)	Missense	25.2	0.0012	.
c.693dup; p.Met232HisfsTer9	NA	48467345; G:GC	DUP	P	Frameshift	27.8	.	.
c.739G>C; p.Ala247Pro	rs112741962	48467394; G:C	SNV	VUS	Missense	22.9	0.0119	0.0400
c.812_813insAA; p.Asp272ArgfsTer6	NA	48467465; C:CAA	INS	NA	Frameshift	23.0	.	.
c.839del; p.Gly280GlufsTer18	rs1419105423	48467492; TG:T	DEL	NA	Frameshift	23.2	.	.
c.856_865dup; p.Ala289GlyfsTer39	rs2040385695	48467509; A:AGGGGCT GCTG	DUP	P	Frameshift	24.6	.	.
c.858del; p.Leu287CysfsTer11	NA	48467509; AG:A	DEL	LP	Frameshift	23.7	.	.
c.868_885del; p.Pro290_Ala295del	rs79318303	48467513; GCTGCTGGCCC CACTGGGT:G	DEL	P/LP	Inframe deletion	15.8	0.0064	.
c.869C>T; p.Pro290Leu	rs148833270	48467524; C:T	SNV	VUS	Missense	21.9	0.0064	.
c.907A>C; p.Thr303Pro	rs76224909	48467562; A:C	SNV	Conflicting: P (1); VUS (1)	Missense	23.7	0.0020	.

HGVS coding DNA; protein	rsid	Position; Ref:Alt	Type	Pathogenicity	Consequence	CADD (scaled)	Allele frequency (%)	
							gnomAD	1000GP
c.914A>G; p.Tyr305Cys	rs370504038	48467569; A:G	SNV	VUS	Missense	25.5	0.0120	.
c.917G>C; p.Gly306Ala	rs780022923	48467572; G:C	SNV	VUS	Missense	18.4	0.0004	.

Pathogenicity refers to the American College of Medical Genetics and Genomics (ACMG) classification (P, pathogenic; LP, likely pathogenic; VUS, variant of uncertain significance; LB, likely benign; B, benign) as per ClinVar or the Leiden Open source Variation Database (LOVD) v3.0 if not in ClinVar. Column "Position; Ref:Alt" refers to genomic coordinates as VCF entry fields. Consequence refers to the most severe translational consequence on Ensembl. Dots in allele frequency columns refer to alleles absent in the database.

Abbreviations: 1000GP, 1000 Genomes Project; Alt, alternate allele; CADD, Combined Annotation-Dependent Depletion; DEL, deletion; DUP, duplication; gnomAD, genome Aggregation Database exome; HGVS, Human Genome Variation Society; INS, insertion; Ref, reference allele; SNV, single nucleotide variant.

Supplementary Table 9. Association of MIRO scores at baseline with incident diagnoses in UK Biobank.

**MIRO score**	**Systemic lupus erythematosus**		**Multiple sclerosis**	
	**HR (95% CI)**	**p-value**	**HR (95% CI)**	**p-value**
**Continuous**				
**Per +1 SD**	**1.66 (1.50, 1.83)**	**<0.0001**	0.97 (0.77, 1.21)	0.756
**Quartiles (vs Q1)**				
**Q2**	1.71 (0.86, 3.40)	0.126	0.81 (0.45, 1.48)	0.498
**Q3**	1.83 (0.93, 3.60)	0.082	0.76 (0.41, 1.42)	0.391
**Q4**	**4.41 (2.40, 8.10)**	**<0.0001**	0.78 (0.42, 1.44)	0.422

Bold characters indicate p-value <0.05 (two-sided tests). Abbreviations: CI, confidence interval; HR, hazard ratio; MIRO, Marker of type I Interferon Response in Olink; SD, standard deviation.

Supplementary Table 10. Description of the 35 reported disease-causing *TREX1* variants observed in UK Biobank.

cDNA change; protein change	rsid	Position; Ref:Alt	Effect	Pathogenicity	CADD (scaled)	LOF	Allele frequency (%)			SLE, n/N (%)	MIRO, n mean (SD)
							UKB	gnomAD	1000GP		
c.1A>G; p.Met1Val	rs761165865	48466656; A:G	Start lost	Conflicting: LP (1); VUS (2)	24.2	NA	0.0004	0.0016	.	0/4 (0.0)	NA
c.58dup; p.Glu20GlyfsTer82	rs78300695	48466711; T:TG	Frameshift	P	28.9	HC	0.0021	0.0127	.	0/20 (0.0)	n=2 -0.23 (0.35)
c.144dup; p.Thr49HisfsTer53	rs748914604	48466792; G:GC	Frameshift	P/LP	23.0	HC	0.0053	0.0076	.	0/50 (0.0)	n=8 -0.24 (0.78)
c.150_151del; p.Gln51GlyfsTer50	rs770607110	48466801; CCT:C	Frameshift	P	17.6	HC	0.0001	0.0004	.	0/1 (0.0)	NA
c.152_153del; p.Gln51ArgfsTer50	rs773808155	48466806; CAG:C	Frameshift	P	19.5	HC	0.0004	0.0004	.	0/4 (0.0)	NA
c.197A>G; p.Lys66Arg	rs188508043	48466852; A:G	Missense	VUS	25.7	NA	0.0003	0.0064	0.0200	0/3 (0.0)	NA
c.212_213del; p.Val71GlyfsTer30	rs74689946	48466860; CTG:C	Frameshift	P	27.2	HC	0.0002	0.0012	.	0/2 (0.0)	n=1 -0.86 (NA)
c.218C>T; p.Pro73Leu	rs755919767	48466873; C:T	Missense	Conflicting: LP (1); VUS (5)	25.2	NA	0.0004	0.0024	.	0/4 (0.0)	n=1 0.08 (NA)
c.236_243dup; p.Ser82LeufsTer9	rs1331920811	48466881; G:GCCTGCAGC	Frameshift	P/LP	24.6	HC	0.0025	0.0008	.	0/23 (0.0)	n=4 -0.03 (1.01)
c.243_246del; p.Ser82ArgfsTer5	rs2040335386	48466893; GCAGC:G	Frameshift	P	29.9	HC	0.0001	.	.	0/1 (0.0)	NA
c.290G>A; p.Arg97His	rs200773268	48466945; G:A	Missense	Conflicting: P (2); LP (2); VUS (1)	27.8	NA	0.0017	0.0060	0.0200	0/16 (0.0)	n=2 -0.49 (0.66)
c.294dup; p.Cys99MetfsTer3	rs760594164	48466947; C:CA	Frameshift	P	24.9	HC	0.0003	0.0072	.	0/3 (0.0)	NA
c.340C>T; p.Arg114Cys	rs760838030	48466995; C:T	Missense	Conflicting: LP (3); VUS (1)	28.5	NA	0.0006	0.0008	.	0/6 (0.0)	NA

cDNA change; protein change	rsid	Position; Ref:Alt	Effect	Pathogenicity	CADD (scaled)	LOF	Allele frequency (%)			SLE, n/N (%)	MIRO, n mean (SD)
							UKB	gnomAD	1000GP		
c.341G>A; p.Arg114His	rs72556554	48466996; G:A	Missense	P/LP	29.3	NA	0.0660	0.0208	.	0/619 (0.0)	n=45 -0.09 (0.73)
c.357G>A; p.Trp119Ter	rs747399274	48467012; G:A	Stop gained	P	37.0	HC	0.0007	.	.	0/7 (0.0)	NA
c.365T>C; p.Val122Ala	rs79993407	48467020; T:C	Missense	NA	27.6	NA	0.0001	.	.	0/1 (0.0)	NA
c.366_368dup; p.Ala123dup	rs77371662	48467020; T:TGGC	Inframe insertion	P	20.4	NA	0.0001	.	.	0/1 (0.0)	NA
c.383G>A; p.Arg128His	rs751124579	48467038; G:A	Missense	VUS	22.8	NA	0.0011	0.0008	.	0/10 (0.0)	NA
c.403C>T; p.Gln135Ter	rs746384382|	48467058; C:T	Stop gained	P	36.0	HC	0.0001	.	.	0/1 (0.0)	NA
c.416del; p.Ala139ValfsTer21	rs763229085	48467070; GC:G	Frameshift	P	26.4	HC	0.0098	0.0028	.	0/92 (0.0)	n=11 0.12 (1.28)[a]
c.473C>T; p.Ala158Val	rs762011967	48467128; C:T	Missense	VUS	23.8	NA	0.0071	0.0044	.	0/67 (0.0)	n=4 -0.12 (1.10)
c.490C>T; p.Arg164Ter	rs78218009	48467145; C:T	Stop gained	P/LP	34.0	HC	0.0009	.	.	0/8 (0.0)	n=2 -0.39 (0.83)
c.513del; p.His171GlnfsTer9	rs2040356109	48467167; AC:A	Frameshift	P	6.9	HC	0.0011	.	.	0/10 (0.0)	n=1 0.77 (NA)
c.553C>T; p.Arg185Cys	rs145721839	48467208; C:T	Missense	VUS	26.0	NA	0.0003	0.0020	0.0200	0/3 (0.0)	NA
c.599_601dup; p.Asp200dup	rs74556809	48467251; G:GTGA	Inframe insertion	LP	17.0	NA	0.0002	0.0008	.	0/2 (0.0)	NA
c.625_628dup; p.Trp210SerfsTer32	rs78948846	48467277; T:TGTCA	Frameshift	NA	32.0	HC	0.0021	0.0008	.	1/20 (5.0)	n=1 0.84 (NA)[b]
c.635del; p.Pro212HisfsTer65	rs756664985	48467288; AC:A	Frameshift	P/LP	28.9	HC	0.0001	0.0004	.	0/1 (0.0)	NA

cDNA change; protein change	rsid	Position; Ref:Alt	Effect	Pathogenicity	CADD (scaled)	LOF	Allele frequency (%)			SLE, n/N (%)	MIRO, n mean (SD)
							UKB	gnomAD	1000GP		
c.667G>A; p.Ala223Thr	rs766785968	48467322; G:A	Missense	Conflicting: LP (2); VUS (2)	25.2	NA	0.0016	0.0012	.	1/15 (6.7)	n=2 -0.21 (0.62)
c.693dup; p.Met232HisfsTer9	NA	48467345; G:GC	Frameshift	P	27.8	HC	0.0005	.	.	0/5 (0.0)	NA
c.739G>C; p.Ala247Pro	rs112741962	48467394; G:C	Missense	VUS	22.9	NA	0.0066	0.0119	0.0400	0/62 (0.0)	n=15 -0.12 (0.75)
c.868_885del; p.Pro290_Ala295del	rs79318303	48467513; GCTGCTGGCCCCACTGGGT:G	Inframe deletion	P/LP	15.8	NA	0.0095	0.0064	.	1/89 (1.1)	n=8 -0.41 (0.46)
c.869C>T; p.Pro290Leu	rs148833270	48467524; C:T	Missense	VUS	21.9	NA	0.0043	0.0064	.	0/40 (0.0)	NA
c.907A>C; p.Thr303Pro	rs76224909	48467562; A:C	Missense	Conflicting: P (1); VUS (1)	23.7	NA	0.0009	0.0020	.	0/8 (0.0)	n=1 0.45 (NA)
c.914A>G; p.Tyr305Cys	rs370504038	48467569; A:G	Missense	VUS	25.5	NA	0.0018	0.0120	.	0/17 (0.0)	n=2 -1.02 (0.63)
c.917G>C; p.Gly306Ala	rs780022923	48467572; G:C	Missense	VUS	18.4	NA	0.0002	0.0004	.	0/2 (0.0)	NA

[a]11 carriers with available MIRO scores (all heterozygotes). Two have MIRO > 2, both with autoimmunity (MIRO=2.48 with myasthenia gravis; MIRO=2.51 with rheumatoid arthritis) but without SLE.
[b]One carrier with available MIRO score and a diagnosis of SLE.
Pathogenicity refers to the American College of Medical Genetics and Genomics (ACMG) classification (P, pathogenic; LP, likely pathogenic; VUS, variant of uncertain significance; LB, likely benign; B, benign) as per ClinVar or the Leiden Open source Variation Database (LOVD) v3.0 if not in ClinVar. Column "Position; Ref:Alt" refers to genomic coordinates as VCF entry fields. Consequence refers to the most severe translational consequence on Ensembl. Dots in allele frequency columns refer to alleles absent in the database. 'n' in column MIRO refers to the number of participants with MIRO score available. A score was available for 110 carriers (80 non-HC and 30 HC).
Abbreviations: 1000GP, 1000 Genomes Project; Alt, alternate allele; CADD, Combined Annotation-Dependent Depletion; gnomAD, genome Aggregation Database exome; HC, high confidence; LOF, Loss-Of-Function Transcript Effect Estimator; MIRO, Marker of type I Interferon Response in Olink; Ref, reference allele; SD, standard deviation; SLE, systemic lupus erythematosus.

Supplementary Table 11. Association of reported disease-causing *TREX1* variants with outcomes in UK Biobank using SAIGE-GENE+.

Phenotype	All		Without c.341G>A (R114H)		Non-pLOF		pLOF	
	Beta (SE)	p	Beta (SE)	p	Beta (SE)	p	Beta (SE)	p
**Diseases with high interferon signature**								
SjD	-0.026 (0.024)	0.435	-0.040 (0.036)	0.422	-0.022 (0.028)	0.583	-0.040 (0.051)	0.616
Systemic sclerosis	0.007 (0.044)	1.000	-0.040 (0.065)	0.741	0.021 (0.050)	0.810	-0.040 (0.093)	0.839
Dermatomyositis	0.041 (0.057)	0.478	-0.040 (0.084)	0.831	0.066 (0.066)	0.319	-0.040 (0.118)	0.882
SLE	-0.005 (0.025)	0.286	0.034 (0.037)	0.171	-0.017 (0.029)	0.315	0.035 (0.053)	0.246
Any	-0.006 (0.017)	0.864	-0.009 (0.024)	0.682	-0.005 (0.019)	1.000	-0.009 (0.035)	0.641
**Diseases with moderate interferon signature**								
Primary biliary cirrhosis	-0.041 (0.037)	0.418	-0.040 (0.055)	0.669	-0.041 (0.042)	0.472	-0.040 (0.077)	0.786
Coeliac disease	-0.021 (0.014)	0.208	-0.018 (0.021)	0.575	-0.016 (0.016)	0.289	-0.040 (0.031)	0.303
Vasculitis	-0.013 (0.019)	0.685	-0.020 (0.029)	0.699	-0.005 (0.022)	1.000	-0.040 (0.040)	0.478
Vitiligo	-0.021 (0.027)	0.451	0.004 (0.040)	0.637	-0.016 (0.030)	0.342	-0.040 (0.063)	0.715
Rheumatoid arthritis	-0.012 (0.008)	0.260	-0.011 (0.012)	0.560	-0.014 (0.009)	0.193	-0.002 (0.018)	1.000
Type 1 diabetes	0.002 (0.013)	1.000	-0.001 (0.021)	1.000	0.002 (0.015)	1.000	0.001 (0.033)	0.890
Any	-0.012 (0.006)	0.073	-0.012 (0.009)	0.278	-0.012 (0.007)	0.126	-0.013 (0.013)	0.469
**Diseases with lower interferon signature**								
Myasthenia gravis	**0.059 (0.035)**	**0.050**	**0.085 (0.047)**	**0.004**	0.008 (0.045)	1.000	**0.147 (0.058)**	**0.002**
Polymyalgia rheumatica	-0.005 (0.012)	0.819	-0.009 (0.018)	0.745	-0.004 (0.014)	0.871	-0.010 (0.025)	0.691
Sarcoidosis	-0.041 (0.021)	0.076	-0.04 (0.031)	0.327	-0.041 (0.023)	0.113	-0.04 (0.046)	0.557
Inflammatory bowel disease	0.000 (0.013)	1.000	-0.007 (0.015)	0.831	0.005 (0.011)	0.812	-0.017 (0.022)	0.625
Hashimoto’s thyroiditis	-0.009 (0.024)	0.167	0.024 (0.034)	0.056	-0.041 (0.028)	0.212	**0.077 (0.046)**	**0.017**
Ankylosing spondylitis	0.022 (0.019)	0.382	**0.059 (0.028)**	**0.045**	0.026 (0.021)	0.321	0.004 (0.043)	0.901
Psoriasis	-0.009 (0.007)	0.216	0.000 (0.010)	0.728	-0.007 (0.008)	0.183	-0.014 (0.016)	0.559
Pernicious anaemia	-0.011 (0.014)	0.606	-0.018 (0.021)	0.582	-0.003 (0.016)	1.000	-0.041 (0.030)	0.275
Graves’ disease	-0.008 (0.010)	0.592	-0.023 (0.015)	0.213	-0.003 (0.011)	1.000	-0.029 (0.022)	0.309

Phenotype		All		Without c.341G>A (R114H)		Non-pLOF		pLOF	
		**Beta (SE)**	**p**	**Beta (SE)**	**p**	**Beta (SE)**	**p**	**Beta (SE)**	**p**
Multiple sclerosis		-0.015 (0.018)	0.593	-0.021 (0.028)	0.650	-0.020 (0.021)	0.487	0.002 (0.044)	1.000
Addison's disease		-0.015 (0.032)	0.806	-0.040 (0.049)	0.610	-0.008 (0.036)	1.000	-0.040 (0.069)	0.745
	Any	-0.006 (0.004)	0.261	-0.006 (0.007)	0.541	-0.005 (0.005)	0.513	-0.012 (0.01)	0.319
**Neurological comorbidity of lupus**									
Depression		0.000 (0.011)	1.000	-0.003 (0.006)	0.809	0.000 (0.005)	1.000	-0.002 (0.009)	0.579
Epilepsy		0.000 (inf)	1.000	-0.001 (0.015)	1.000	0.001 (0.011)	1.000	-0.005 (0.022)	1.000
Ischemic stroke		0.005 (0.007)	0.657	0.009 (0.011)	0.237	0.011 (0.008)	0.259	-0.015 (0.015)	0.495
Migraine		0.000 (0.006)	0.658	-0.008 (0.009)	0.399	0.002 (0.006)	0.640	-0.012 (0.013)	0.512
Psychosis		-0.010 (0.025)	0.835	-0.005 (0.038)	0.838	-0.003 (0.028)	0.770	-0.040 (0.055)	0.649
Vascular dementia		-0.002 (0.018)	0.120	0.038 (0.027)	0.105	-0.015 (0.022)	0.155	0.033 (0.038)	0.564
	Any	-0.001 (0.003)	0.812	-0.005 (0.005)	0.472	0.001 (0.004)	0.862	-0.008 (0.007)	0.310
**Neuroradiological phenotypes**									
Hippocampal grey matter volume		0.002 (0.004)	0.382	0.000 (0.002)	0.081	-0.001 (0.005)	0.620	0.015 (0.010)	0.070
Total brain volume		-0.004 (0.004)	0.423	-0.004 (0.006)	0.506	-0.005 (0.004)	0.335	0.000 (0.001)	0.713
WMH volume		-0.002 (0.004)	0.752	-0.005 (0.007)	0.388	-0.003 (0.005)	0.598	0.003 (0.010)	0.746
**Proteomic score**									
MIRO		-0.002 (0.005)	0.802	-0.004 (0.007)	0.582	-0.003 (0.006)	0.692	0.001 (0.008)	0.910

Set-based rare variant tests implemented in SAIGE-GENE+ using burden tests to generate effect sizes and SKAT-O to generate p-values (two-sided tests). Bold characters indicate p <0.05 (nominal significance). Abbreviations: MAF, minor allele frequency; MIRO, Marker of type I Interferon Response in Olink; p, p-value; pLOF, high-confidence predicted loss-of-function; SE, standard error; SjD, Sjogren disease; SKAT-O, sequence kernel association test - optimal; SLE, systemic lupus erythematosus; WMH, white matter hyperintensity.

Supplementary Table 12. Descriptive analysis of systemic lupus erythematosus/Sjogren disease cases and type I interferon signature by *TREX1* genotype.

Ancestry	Carriers, n (%)	Systemic lupus erythematosus or Sjogren disease						MIRO score		
		Any source of diagnosis			Hospital records only					
		Cases, n (%)	Non-carriers, n (%)	Carriers, n (%)	Cases, n (%)	Non-carriers, n (%)	Carriers, n (%)	n (%)	Non-carriers, n (%); mean (SD)	Carriers, n (%); mean (SD)
European (n=393,433)	933 (0.2)	2,050 (0.5)	2,047 (0.5)	3 (0.3)	1,553 (0.4)	1,551 (0.4)	2 (0.2)	29,361 (7.5)	29,285 (99.7); -0.01 (0.99)	76 (0.3); -0.07 (0.85)
South Asian (n=6,429)	28 (0.4)	77 (1.2)	77 (1.2)	0 (0.0)	59 (0.9)	59 (0.9)	0 (0.0)	435 (6.8)	433 (99.5); 0.55 (1.22)	2 (0.5); -0.23 (0.35)
West African (n=3,854)	17 (0.4)	40 (1.0)	40 (1.0)	0 (0.0)	32 (0.8)	32 (0.8)	0 (0.0)	457 (11.9)	454 (99.3); 0.23 (1.03)	3 (0.7); 0.11 (0.35)
East Asian (n=1,787)	4 (0.2)	13 (0.7)	13 (0.7)	0 (0.0)	12 (0.7)	12 (0.7)	0 (0.0)	127 (7.1)	127 (100.0); -0.25 (0.91)	0 (0.0); NA

Descriptive results are presented without statistical tests due to sparse data yielding unstable estimates in non-European ancestry groups. For lupus/Sjogren, columns "Non-carriers" and "Carriers" refer to the number of participants by *TREX1* genotype with any of the two diseases. For MIRO, column "n (%)" represents the number of participants with MIRO available over the number of participants within the ancestry group and columns "Non-carriers" and "Carriers" represent the number of participants by *TREX1* genotype with MIRO available. Abbreviations: MIRO, Marker of type I Interferon Response in Olink.

Supplementary Table 13. Search strategy in Ovid EMBASE and Ovid MEDLINE.

Line	Entry	Record count
**EMBASE**		
**Genetic variants concept**		
1	(snp* or snv* or polymorphism* or mutation* or variant* or homozygo* or heterozygo* or genome*wide or gwas).mp	2,428,378
2	Exp gene mutation/ or Exp genetic association/	1,158,583
3	or/ 1-2	2,622,283
***TREX1* concept**		
4	(trex1 or three prime repair exonuclease 1 or crv or ags1 or drn3 or herns or rvcls).mp	1,661
**Systemic lupus erythematosus concept**		
5	(lupus or sle).mp	197,091
6	Exp lupus erythematosus/ or Exp systemic lupus erythematosus/	157,315
7	or/ 5-6	197,093
**Combine concepts**		
8	3 and 4 and 7	232
9	8 not ((exp animal/ or nonhuman/) not exp human/)	200
10	9 not (case study/ or case report/)	163
**MEDLINE**		
**Genetic variants concept**		
1	(snp* or snv* or polymorphism* or mutation* or variant* or homozygo* or heterozygo* or genome*wide or gwas).mp	1,568,473
2	Exp mutation/ or Exp genetic association studies/ or Exp genetic predisposition to disease/	1,035,428
3	or/ 1-2	1,826,587
***TREX1* concept**		
4	(trex1 or three prime repair exonuclease 1 or crv or ags1 or drn3 or herns or rvcls).mp	909
**Systemic lupus erythematosus concept**		
5	(lupus or sle).mp	98,768
6	Exp lupus erythematosus, systemic/	69,289
7	or/ 5-6	98,768
**Combine concepts**		
8	3 and 4 and 7	88
9	8 not ((exp animal/ or nonhuman/) not exp human/)	84
10	9 not (case study/ or case report/)	67

Abbreviations: exp, explode; mp, multi-purpose fields.

Supplementary Table 14. Description of studies included in the systematic review.

Characteristic	Lee-Kirsch *et al*, 2007	Namjou *et al*, 2011	Jiang *et al*, 2019
**Journal**	*Nature Genetics*	*Genes and Immunity*	*Nature Communications*
**Cases**			
**Definition**	Diagnostic criteria of the American Rheumatism Association	4 of the 11 revised 1997 American College of Rheumatology criteria	Not reported
**Source**	**UK cases (n=218)**: participants from the UK (unclear); **German cases (n=199)**: participants in the Genetic risk factors of SLE study (unclear); **Finnish cases (n=188)**: children in nuclear family trios (patients who attended the University Hospitals of Helsinki and Kuopio between 1992 and 1995 [phase 1] or the central hospitals and 4 other major hospitals in Finland between 1993 and 1996 [phase 2])	Cases and controls enrolled in the Lupus Family Registry and the Repository and Lupus Genetics Studies at the Oklahoma Medical Research Foundation, and by collaborators on 4 other studies	Participants in the Australian Point Mutation in Systemic Lupus Erythematosus study (Australia, 2008-2014 [cohort 1] and 2015-2017 [cohort 2])
**Demographics**	Not reported	**Sex**: male, 8.4%; female, 91.6%	**Age:** pediatric, 14%; adolescent, 26%; adult, 55%; rest unknown; **Sex:** male, 20.3%; female, 79.7%
**Ancestry**	**UK cases (n=218)**: 'Caucasian': 85.8%; Indian: 7.1%; Afro-Caribbean: 4.5%; Oriental, mixed or Filipino: 2.6%; **Other cohorts of cases**: not reported	European-American: n=3,936 (47.0%); Asian: n=1,265 (15.1%); African-American: n=1,527 (18.2%); Gullah: n=152 (1.8%); Hispanic (others): n=1,492 (17.8%)	European, 79.7%
**Controls**			
**Source**	**UK controls (n=200)**: participants in the Genetic mechanisms of cardiovascular disease cohort at the University of Glasgow; **German controls (n=1512)**: blood donation program at the Medizinische Hochschule Hannover and the Universitätsklinikum Dresden; **Finnish controls (n=376)**: parents of SLE cases in nuclear family trios.	Cases and controls enrolled in the Lupus Family Registry and the Repository and Lupus Genetics Studies at the Oklahoma Medical Research Foundation, and by collaborators on 4 other studies.	Participants in the 45 and Up cohort (Australia, 2005-2009) and the ASPREE study (Australia & US, 2010-2014)
**Demographics**	Not reported	**Sex**: male, 26.4%; female, 73.6%	Age ≥45 (45 and Up) or ≥65 (ASPREE)
**Ancestry**	Not reported	European-American: n=3,491 (46.6%); Asian: n=1,260 (16.8%); African American: n=1,811 (24.2%); Gullah: n=123 (1.6%); Hispanic (others): n=807 (10.8%)	European, 100%

Characteristic	Lee-Kirsch *et al*, 2007	Namjou *et al*, 2011	Jiang *et al*, 2019
**Sequencing technique and QC**	Genomic DNA extracted from peripheral lymphocytes. Amplification of the entire coding exon and part of the 3'UTR of *TREX1* with 3 overlapping PCR amplicons. Purified PCR products then sequenced using fluorescent dye terminator chemistry on ABI 3730 instruments. All identified variants were re-sequenced and validated independently by pyrosequencing.	Project part of the Lupus Large Association Study. Genotype calls generated using the Illumina iSelect technology, and SNP clusters evaluated with the Illumina BEADSTUDIO(r) software package. Filtering for genotypic data from samples with call rate >90% and from SNPs with a call frequency >90%. Additional variant-level QC included Hardy Weinberg proportions with p>0.01 in controls and >0.0001 in cases, total proportion missing <5%, p>0.05 for differential missingness between cases and controls. Exclusion of samples with <90% call rate or heterogeneity >5 SD around the mean. Genetic outliers determined by principal component analysis were removed.	Whole-genome sequencing (for controls and 12.7% of cases) or whole-exome sequencing (87.3% of cases). DNA samples enriched with the Human SureSelect XT2 All Exon V4 kit and sequenced by Illumina HiSeq 2000. Principal variance component analysis (PVCA) was conducted to identify technical variability and batch correct.
**Test**	Comparison of the number of variants in cases and controls with two-sided Fisher's exact tests	Test for association completed in PLINK for each SNP to generate odds ratios and q values	No test performed for *TREX1* (frequencies reported only)
**Control for population stratification**	No	Yes (exclusion of genetic outliers as per genetic principal components)	Not in the reported results for *TREX1* (correction for population stratification using rADMIXTURE in primary analyses)
**Variants included**	Nonsynonymous variants	Genotyping of 40 SNPs in the *TREX1* genomic region, including previously reported rare SNPs and more common tag SNPs.	Rare (MAF <0.005) missense and splice site variants
**Variants observed**			
**Cases**	c.341G>A (p.Arg114His), n=1; c.473C>T (p.Ala158Val), n=1; c.635del (p.Pro212HisfsTer65), n=1; c.679G>A (p.Gly227Ser) & c.739G>C (p.Ala247Pro), n=1; c.720G>C (p.Arg240Ser), n=1; c.797A>G (p.Glu266Gly), n=2; c.811_812dup (p.Asp272ArgfsTer6), n=1; c.869C>T (p.Pro290Leu), n=1; c.914A>G (p.Tyr305Cys), n=1; c.917G>C (p.Gly306Ala) (in Sjogren), n=1; c.979delC (3'UTR), n=1	c.341G>A (p.Arg114His), n=13; c.720G>T (p.Arg240Ser), n=11; c.739G>C (p.Ala247Pro), n=3; c.797A>G (p.Glu266Gly), n=27; c.914A>G (p.Tyr305Cys), n=5	Not reported
**Controls**	c.797A>G (p.Glu266Gly), n=2	c.341G>A (p.Arg114His), n=6; c.720G>T (p.Arg240Ser), n=12; c.739G>C (p.Ala247Pro), n=10; c.797A>G (p.Glu266Gly), n=19	Not reported

Abbreviations: MAF, minor allele frequency; QC, quality control; SNP, single nucleotide polymorphism.

Supplementary Table 15. Risk of bias assessment (Q-Genie) for studies included in the systematic review.

**Criteria**	**Lee-Kirsch *et al*, 2007**	**Namjou *et al*, 2011**	**Jiang *et al*, 2019**
1. Rationale for study	5.5	5.5	5.5
2. Selection and definition of outcome of interest	3.5	4.5	3.5
3. Selection and comparability of comparison groups	3.0	4.5	2.5
4. Technical classification of the exposure	3.0	4.5	3.5
5. Non-technical classification of the exposure	2.5	4.0	2.5
6. Other sources of bias	3.5	4.0	4.0
7. Sample size and power	4.0	4.0	4.0
8. A priori planning of analyses	2.5	2.5	4.0
9. Statistical methods and control for confounding	1.5	4.5	3.0
10. Testing of assumptions and inferences for genetic analyses	3.5	4.0	3.0
11. Appropriateness of inferences drawn from results	4.0	3.5	4.0
**Overall**	**36.5 (moderate quality)**	**45.5 (good quality)**	**39.5 (moderate quality)**

Scores obtained after averaging independent ratings from two reviewers. Overall scoring guidance for studies with control groups in Q-Genie: scores ≤35 indicate poor quality studies, >35 and ≤45 indicate studies of moderate quality, and >45 indicate good quality studies.

Supplementary Table 16. Weights for each protein included in the MIRO score defined from their β coefficient in the penalized logistic regression.

Protein	UniProt entry	Weight
SIGLEC1	Q9BZZ2	0.287
RIG-I (DDX58)	O95786	0.254
IFIT3	O14879	0.097

Abbreviations: MIRO, Marker of type I Interferon Response in Olink.

SUPPLEMENTARY NOTES

Supplementary Note 1. Checklist for the STrengthening the REporting of Genetic Association studies (STREGA) reporting recommendations, extended from STROBE Statement.

Item	Item no	STROBE Guideline	Extension for Genetic Association Studies (STREGA)	Page no
**Title and Abstract**	1	(a) Indicate the study's design with a commonly used term in the title or the abstract.		Abstract
		(b) Provide in the abstract an informative and balanced summary of what was done and what was found.		Abstract
**Introduction**				
Background rationale	2	Explain the scientific background and rationale for the investigation being reported.		3
Objectives	3	State specific objectives, including any pre-specified hypotheses	***State if the study is the first report of a genetic association, a replication effort, or both.***	3
**Methods**				
Study design	4	Present key elements of study design early in the paper.		18, 22
Setting	5	Describe the setting, locations and relevant dates, including periods of recruitment, exposure, follow-up and data collection.		18
Participants	6	(a) **Cohort study** – Give the eligibility criteria, and the sources and methods of selection of participants. Describe methods of follow-up.<br><br>**Case–control study** – Give the eligibility criteria, and the sources and methods of case ascertainment and control selection. Give the rationale for the choice of cases and controls.<br><br>**Cross-sectional study** – Give the eligibility criteria, and the sources and methods of selection of participants.	***Give information on the criteria and methods for selection of subsets of participants from a larger study, when relevant.***	18

		**(b) Cohort study** – For matched studies, give matching criteria and number of exposed and unexposed.<br><br>**Case–control study** – For matched studies, give matching criteria and the number of controls per case.		
Variables	7	(a) Clearly define all outcomes, exposures, predictors, potential confounders, and effect modifiers. Give diagnostic criteria, if applicable.	***(b) Clearly define genetic exposures (genetic variants) using a widely –used nomenclature system. Identify variables likely to be associated with population stratification (confounding by ethnic origin).***	20, 28
Data sources measurement	8*	(a) For each variable of interest, give sources of data and details of methods of assessment (measurement). Describe comparability of assessment methods if there is more than one group.	***(b) Describe laboratory methods, including source and storage of DNA, genotyping methods and platforms (including the allele calling algorithm used, and its version), error rates and call rates. State the laboratory /centre where genotyping was done. Describe***	19-20

			comparability of laboratory methods if there is more than one group. Specify whether genotypes were assigned using all of the data from the study simultaneously or in smaller batches.	
Bias	9	(a) Describe any efforts to address potential sources of bias.	***(b) For quantitative outcome variables, specify if any investigation of potential bias resulting from pharmacotherapy was undertaken. If relevant, describe the nature and magnitude of the potential bias, and explain what approach was used to deal with this.***	28
Study size	10	Explain how the study size was arrived at.		28
Quantitative variables	11	Explain how quantitative variables were handled in the analyses. If applicable, describe which groupings were chosen, and why.	***If applicable, describe how effects of treatment were dealt with.***	18
Statistical methods	12	(a) Describe all statistical methods, including those used to control for confounding.	***State software version used and options***	28-29

			(or settings) chosen.	
		(b) Describe any methods used to examine subgroups and interactions.		28
		(c) Explain how missing data were addressed.		
		(d) **Cohort study –** If applicable, explain how loss to follow-up was addressed.<br><br>**Case–control study –** If applicable, explain how matching of cases and controls was addressed.<br><br>**Cross-sectional study –** If applicable, describe analytical methods taking account of sampling strategy.		
		(e) Describe any sensitivity analyses.		
			(f) State whether Hardy-Weinberg equilibrium was considered and, if so, how.	21
			(g) Describe any methods used for inferring genotypes or haplotypes.	NA
			(h) Describe any methods used to assess or address population stratification.	28
			(i) Describe any methods used to address multiple comparisons or to control risk of false	29

			positive findings.	
			(j) Describe any methods used to address and correct for relatedness among subjects.	28-29
**Results**				
Participants	13*	(a) Report the numbers of individuals at each stage of the study – e.g. numbers potentially eligible, examined for eligibility, confirmed eligible, included in the study, completing follow-up and analysed.	***Report numbers of individuals in whom genotyping was attempted and numbers of individuals in whom genotyping was successful.***	20
		(b) Give reasons for non-participation at each stage.		
		(c) Consider use of a flow diagram.		
Descriptive data	14*	(a) Give characteristics of study participants (e.g. demographic, clinical, social) and information on exposures and potential confounders.	***Consider giving information by genotype.***	20, ST3
		(b) Indicate the number of participants with missing data for each variable of interest.		
		(c) **Cohort study** – Summarize follow-up time, e.g. average and total amount.		
Outcome data	15*	**Cohort study** – Report numbers of outcome events or summary measures over time.	***Report outcomes (phenotypes) for each genotype category over time***	5, 8-9
		**Case–control study** – Report numbers in each exposure category, or summary measures of exposure.	***Report numbers in each genotype category***	

		**Cross-sectional study –** Report numbers of outcome events or summary measures.	***Report outcomes (phenotypes) for each genotype category***	
Main results	16	(a) Give unadjusted estimates and, if applicable, confounder-adjusted estimates and their precision (e.g. 95% confidence intervals). Make clear which confounders were adjusted for and why they were included.		5, 8-9
		(b) Report category boundaries when continuous variables were categorized.		
		(c) If relevant, consider translating estimates of relative risk into absolute risk for a meaningful time period.		
			(d) Report results of any adjustments for multiple comparisons.	5, 8-9
Other analyses	17	(a) Report other analyses done – e.g. analyses of subgroups and interactions, and sensitivity analyses.		9
			(b) If numerous genetic exposures (genetic variants) were examined, summarize results from all analyses undertaken.	9
			(c) If detailed results are available elsewhere, state how they can be accessed.	NA
**Discussion**				
Key results	18	Summarize key results with reference to study objectives.		12
Limitations	19	Discuss limitations of the study, taking into account sources of potential bias or		15-16

		imprecision. Discuss both direction and magnitude of any potential bias.	
Interpretation	20	Give a cautious overall interpretation of results considering objectives, limitations, multiplicity of analyses, results from similar studies, and other relevant evidence.	12-17
Generalizability	21	Discuss the generalizability (external validity) of the study results.	14
**Other information**			
Funding	22	Give the source of funding and the role of the funders for the present study and, if applicable, for the original study on which the present article is based.	36

Supplementary Note 2. Checklist for the Preferred Reporting Items for Systematic Reviews and Meta-analyses (PRISMA) statement.

Section and Topic	Item #	Checklist item	Location where item is reported
**TITLE**			
Title	1	Identify the report as a systematic review.	p10
**ABSTRACT**			
Abstract	2	See the PRISMA 2020 for Abstracts checklist.	NA
**INTRODUCTION**			
Rationale	3	Describe the rationale for the review in the context of existing knowledge.	p3
Objectives	4	Provide an explicit statement of the objective(s) or question(s) the review addresses.	p10
**METHODS**			
Eligibility criteria	5	Specify the inclusion and exclusion criteria for the review and how studies were grouped for the syntheses.	p26
Information sources	6	Specify all databases, registers, websites, organisations, reference lists and other sources searched or consulted to identify studies. Specify the date when each source was last searched or consulted.	p26
Search strategy	7	Present the full search strategies for all databases, registers and websites, including any filters and limits used.	ST13
Selection process	8	Specify the methods used to decide whether a study met the inclusion criteria of the review, including how many reviewers screened each record and each report retrieved, whether they worked independently, and if applicable, details of automation tools used in the process.	p27
Data collection process	9	Specify the methods used to collect data from reports, including how many reviewers collected data from each report, whether they worked independently, any processes for obtaining or confirming data from study investigators, and if applicable, details of automation tools used in the process.	p27
Data items	10a	List and define all outcomes for which data were sought. Specify whether all results that were compatible with each outcome domain in each study were sought (e.g. for all measures, time points, analyses), and if not, the methods used to decide which results to collect.	pp27-28
	10b	List and define all other variables for which data were sought (e.g. participant and intervention characteristics, funding sources). Describe any assumptions made about any missing or unclear information.	pp27-28
Study risk of bias assessment	11	Specify the methods used to assess risk of bias in the included studies, including details of the tool(s) used, how many reviewers assessed each study and whether they worked independently, and if applicable, details of automation tools used in the process.	p27
Effect measures	12	Specify for each outcome the effect measure(s) (e.g. risk ratio, mean difference) used in the synthesis or presentation of results.	p27
Synthesis methods	13a	Describe the processes used to decide which studies were eligible for each synthesis (e.g. tabulating the study intervention characteristics and comparing against the planned groups for each synthesis (item #5)).	pp27-28
	13b	Describe any methods required to prepare the data for presentation or synthesis, such as handling of missing summary statistics, or data conversions.	pp27-28
	13c	Describe any methods used to tabulate or visually display results of individual studies and syntheses.	pp27-28
	13d	Describe any methods used to synthesize results and provide a rationale for the choice(s). If meta-analysis was performed, describe the	pp27-28

Section and Topic	Item #	Checklist item	Location where item is reported
		model(s), method(s) to identify the presence and extent of statistical heterogeneity, and software package(s) used.	
	13e	Describe any methods used to explore possible causes of heterogeneity among study results (e.g. subgroup analysis, meta-regression).	pp27-28
	13f	Describe any sensitivity analyses conducted to assess robustness of the synthesized results.	pp27-28
Reporting bias assessment	14	Describe any methods used to assess risk of bias due to missing results in a synthesis (arising from reporting biases).	p27
Certainty assessment	15	Describe any methods used to assess certainty (or confidence) in the body of evidence for an outcome.	p28
**RESULTS**			
Study selection	16a	Describe the results of the search and selection process, from the number of records identified in the search to the number of studies included in the review, ideally using a flow diagram.	p10
	16b	Cite studies that might appear to meet the inclusion criteria, but which were excluded, and explain why they were excluded.	p10
Study characteristics	17	Cite each included study and present its characteristics.	p10
Risk of bias in studies	18	Present assessments of risk of bias for each included study.	p10
Results of individual studies	19	For all outcomes, present, for each study: (a) summary statistics for each group (where appropriate) and (b) an effect estimate and its precision (e.g. confidence/credible interval), ideally using structured tables or plots.	p11
Results of syntheses	20a	For each synthesis, briefly summarise the characteristics and risk of bias among contributing studies.	p11
	20b	Present results of all statistical syntheses conducted. If meta-analysis was done, present for each the summary estimate and its precision (e.g. confidence/credible interval) and measures of statistical heterogeneity. If comparing groups, describe the direction of the effect.	p11
	20c	Present results of all investigations of possible causes of heterogeneity among study results.	p11
	20d	Present results of all sensitivity analyses conducted to assess the robustness of the synthesized results.	p11
Reporting biases	21	Present assessments of risk of bias due to missing results (arising from reporting biases) for each synthesis assessed.	p11
Certainty of evidence	22	Present assessments of certainty (or confidence) in the body of evidence for each outcome assessed.	p11
**DISCUSSION**			
Discussion	23a	Provide a general interpretation of the results in the context of other evidence.	p11
	23b	Discuss any limitations of the evidence included in the review.	pp15-16
	23c	Discuss any limitations of the review processes used.	pp15-16
	23d	Discuss implications of the results for practice, policy, and future research.	pp15-16
**OTHER INFORMATION**			
Registration and protocol	24a	Provide registration information for the review, including register name and registration number, or state that the review was not registered.	p26

Section and Topic	Item #	Checklist item	Location where item is reported
	24b	Indicate where the review protocol can be accessed, or state that a protocol was not prepared.	p26
	24c	Describe and explain any amendments to information provided at registration or in the protocol.	NA
Support	25	Describe sources of financial or non-financial support for the review, and the role of the funders or sponsors in the review.	p36
Competing interests	26	Declare any competing interests of review authors.	p36
Availability of data, code and other materials	27	Report which of the following are publicly available and where they can be found: template data collection forms; data extracted from included studies; data used for all analyses; analytic code; any other materials used in the review.	p26

SUPPLEMENTARY REFERENCES

1. Conrad, N. *et al.* Autoimmune diseases and cardiovascular risk: a population-based study on 19 autoimmune diseases and 12 cardiovascular diseases in 22 million individuals in the UK. *Lancet* **400**, 733-743 (2022).
