# Supplementary material for: Oligoprotein type I interferon signatures, but not TREX1 variants, increase risk of systemic lupus erythematosus in UK Biobank
Source: Nat Commun. 2026 Jan 27;17:1073. doi: 10.1038/s41467-025-67832-z (PMC12852752; doi:10.1038/s41467-025-67832-z)
Supplement: Supplementary file 2 — Reporting Summary [file 41467_2025_67832_MOESM2_ESM.pdf]

Corresponding author(s): David Hunt, david.hunt@ed.ac.uk
Last updated by author(s): Oct 8, 2025

Reporting Summary

Nature Portfolio wishes to improve the reproducibility of the work that we publish. This form provides structure for consistency and transparency in reporting. For further information on Nature Portfolio policies, see our Editorial Policies and the Editorial Policy Checklist.

Statistics

For all statistical analyses, confirm that the following items are present in the figure legend, table legend, main text, or Methods section.

n/a	Confirmed	
☐	☒	The exact sample size (*n*) for each experimental group/condition, given as a discrete number and unit of measurement
☐	☒	A statement on whether measurements were taken from distinct samples or whether the same sample was measured repeatedly
☐	☒	The statistical test(s) used AND whether they are one- or two-sided<br>*Only common tests should be described solely by name; describe more complex techniques in the Methods section.*
☐	☒	A description of all covariates tested
☐	☒	A description of any assumptions or corrections, such as tests of normality and adjustment for multiple comparisons
☐	☒	A full description of the statistical parameters including central tendency (e.g. means) or other basic estimates (e.g. regression coefficient) AND variation (e.g. standard deviation) or associated estimates of uncertainty (e.g. confidence intervals)
☐	☒	For null hypothesis testing, the test statistic (e.g. *F*, *t*, *r*) with confidence intervals, effect sizes, degrees of freedom and *P* value noted<br>*Give P values as exact values whenever suitable.*
☒	☐	For Bayesian analysis, information on the choice of priors and Markov chain Monte Carlo settings
☒	☐	For hierarchical and complex designs, identification of the appropriate level for tests and full reporting of outcomes
☐	☒	Estimates of effect sizes (e.g. Cohen's *d*, Pearson's *r*), indicating how they were calculated

Our web collection on statistics for biologists contains articles on many of the points above.

Software and code

Policy information about availability of computer code

Data collection	No software for data collection.
Data analysis	Analyses were performed on the UKB Research Analysis Platform using JupyterLab (v3.6.1) with Hail (v0.2.78), Python (v3.9.16) and R (v4.2.0). Logistic and Cox regressions were conducted on R using logistf (v1.26.0) and survival (v3.7.0), and meta-analyses were conducted using metafor (v4.6-0). Visualization of genomic and phenotype data was conducted on R using trackViewer, UpSetR (v1.4.0), forestploter (v1.1.1) and ggplot2 (v.3.4.4).

For manuscripts utilizing custom algorithms or software that are central to the research but not yet described in published literature, software must be made available to editors and reviewers. We strongly encourage code deposition in a community repository (e.g. GitHub). See the Nature Portfolio guidelines for submitting code & software for further information.

Data

Policy information about availability of data

All manuscripts must include a data availability statement. This statement should provide the following information, where applicable:

- Accession codes, unique identifiers, or web links for publicly available datasets
- A description of any restrictions on data availability
- For clinical datasets or third party data, please ensure that the statement adheres to our policy

All data are included in the Supplementary Information or available from the authors, as are unique reagents used in this Article. The raw numbers for charts and graphs are available in the Source Data file whenever possible. UK Biobank data supporting the findings can be accessed through application to the resource:

https://www.ukbiobank.ac.uk/enable-your-research/apply-for-access.

Research involving human participants, their data, or biological material

Policy information about studies with human participants or human data. See also policy information about sex, gender (identity/presentation), and sexual orientation and race, ethnicity and racism.

Reporting on sex and gender	We used reported and genetically determined sex (from array data) in our study. These were used to describe the cohort by disease and genotype, and to adjust models for the potential confounding effect of sex.
Reporting on race, ethnicity, or other socially relevant groupings	We determined ethnic background from self-reports at baseline. For genetic analyses, genetic ancestry groups were defined from genetic principal components. These groups were used to perform analyses in participants of different ancestries. Details are provided in the Methods section.
Population characteristics	UK Biobank (UKB) is a large population-based study of 502k volunteers recruited through UK patient registries. Cohort characteristics are presented in Supplementary Tables 1 & 3. UKPSSR is a national observational cohort of Sjogren disease patients fulfilling the 2002 American European Consensus Group classification criteria and recruited across 30 UK centres.
Recruitment	UKB is a large population-based cohort of 502k volunteers aged 40-69 years at recruitment (2006-2010) who were identified through UK patient registries (response rate: 5.5%). Participants in UKPSSR were recruited based on the 2002 American European Consensus Group classification criteria across 30 UK centres.
Ethics oversight	UKB has received ethical approval from the North West Multicentre Research Ethics Committee, and all participants provided written informed consent at recruitment. Participants of UKPSSR gave written consent for collection of clinical and peripheral blood samples and the research ethics approval was granted by the UK National Research Ethics Committee North West – Haydock

Note that full information on the approval of the study protocol must also be provided in the manuscript.

Field-specific reporting

Please select the one below that is the best fit for your research. If you are not sure, read the appropriate sections before making your selection.

[x] Life sciences [ ] Behavioural & social sciences [ ] Ecological, evolutionary & environmental sciences

For a reference copy of the document with all sections, see nature.com/documents/nr-reporting-summary-flat.pdf

Life sciences study design

All studies must disclose on these points even when the disclosure is negative.

Sample size	We used the whole of UK Biobank for this analysis and did not perform a pre-specified power calculation. UKPSSR samples were restricted to those with proteomics and transcriptomics data.
Data exclusions	Exclusions in UKB were based on low-quality calls from exome sequencing and are described in the Methods (Whole-exome sequencing section).
Replication	We performed a systematic review and meta-analysis of published evidence to contrast our results with those of previous experiments.
Randomization	Randomization was not performed, but potential confounders were accounted for in the analyses.
Blinding	We did not apply blinding of the samples for analyses.

Reporting for specific materials, systems and methods

We require information from authors about some types of materials, experimental systems and methods used in many studies. Here, indicate whether each material, system or method listed is relevant to your study. If you are not sure if a list item applies to your research, read the appropriate section before selecting a response.

Materials & experimental systems

n/a	Involved in the study
x	Antibodies
	x Eukaryotic cell lines
x	Palaeontology and archaeology
x	Animals and other organisms
x	Clinical data
x	Dual use research of concern
x	Plants

Methods

n/a	Involved in the study
x	ChIP-seq
x	Flow cytometry
x	MRI-based neuroimaging

Eukaryotic cell lines

Policy information about cell lines and Sex and Gender in Research

Cell line source(s)	These cells were prepared from TREX1-/- embryos. They were a kind gift from Martin Reijns and Andrew Jackson.
Authentication	Absence of TREX1 expression was authenticated by RT-PCR and loss-of-function was confirmed by nuclease assay.
Mycoplasma contamination	Not tested
Commonly misidentified lines (See ICLAC register)	N/A

Plants

Seed stocks	*Report on the source of all seed stocks or other plant material used. If applicable, state the seed stock centre and catalogue number. If plant specimens were collected from the field, describe the collection location, date and sampling procedures.*
Novel plant genotypes	*Describe the methods by which all novel plant genotypes were produced. This includes those generated by transgenic approaches, gene editing, chemical/radiation-based mutagenesis and hybridization. For transgenic lines, describe the transformation method, the number of independent lines analyzed and the generation upon which experiments were performed. For gene-edited lines, describe the editor used, the endogenous sequence targeted for editing, the targeting guide RNA sequence (if applicable) and how the editor was applied.*
Authentication	*Describe any authentication procedures for each seed stock used or novel genotype generated. Describe any experiments used to assess the effect of a mutation and, where applicable, how potential secondary effects (e.g. second site T-DNA insertions, mosiacism, off-target gene editing) were examined.*
